# An efficient route to the synthesis of novel zwitterionic pyridinium-cyanopropenides with 3-heteroaryl-substituted trimethinium salts†

**DOI:** 10.1039/d2ra02465a

**Published:** 2022-05-30

**Authors:** Ziba Rafiee Samani, Abdolmohammad Mehranpour

**Affiliations:** Department of Chemistry, Faculty of Sciences, Persian Gulf University Bushehr 75169 Iran ammehranpour@hotmail.com

## Abstract

In this study, eight new zwitterionic derivatives were synthesized using a simple design method from the reaction of various 2-substituted 1,3-bis(dimethylamino)-trimethinium salts with malononitrile or ethyl 2-cyanoacetate in excellent yields in the presence of triethylamine in ethanol at reflux. The molecular structures of the new compounds were confirmed by IR, UV/vis, mass, ^1^H, and ^13^C NMR spectra as well as by elemental analyses.

## Abstract 2

Synthesis of new zwitterionic derivatives *via* the reaction of 2-substituted 1,3-bis(dimethylamino)-trimethinium salts with malononitrile or ethyl cyanoacetate in the presence of Et_3_N in ethanol at reflux.

## Graphical abstract

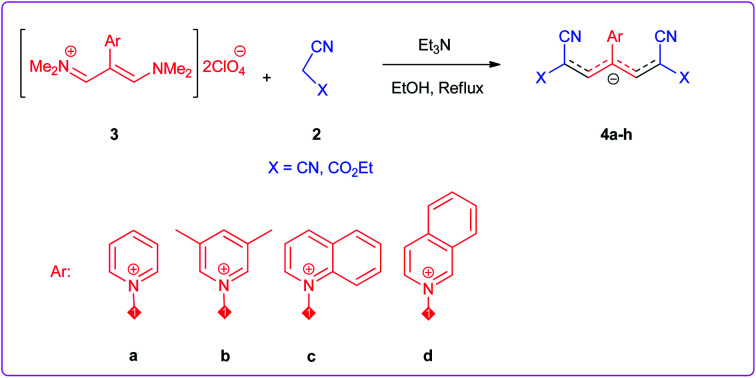

## Introduction

Polymethine dyes constitute an independent class of conjugated π systems different from polyenes and aromatics. Polymethinic π systems are conjugated planar open-chain or ring compounds of the general formula 1 with high polarizabilities and medium-sized delocalization energies, with equal π-bond orders but unequal (alternating) π-electron densities along the carbon chain as well as relatively high chemical reactivity, preferring substitution over addition reactions.^1,2^ Polymethine dyes have unique inherent properties include conjugated structure, relatively good stability, medium fluorescence intensities, high molar absorption coefficients (about 10^5^ dm^3^ mol^−1^ cm^−1^), and narrow bandwidths invisible region.^3,4^

Cyanine dyes are compounds that have a wide range of applications in many fields of science, pharmacology, medicine, and technology engineering. Such as bactericidal and fungicidal, anti-cancer, acid–base indicators, laser technology, organic solar cells, dyes for polymers, and spectral sensitizers for silver halide emulsion, *etc.*^5–14^
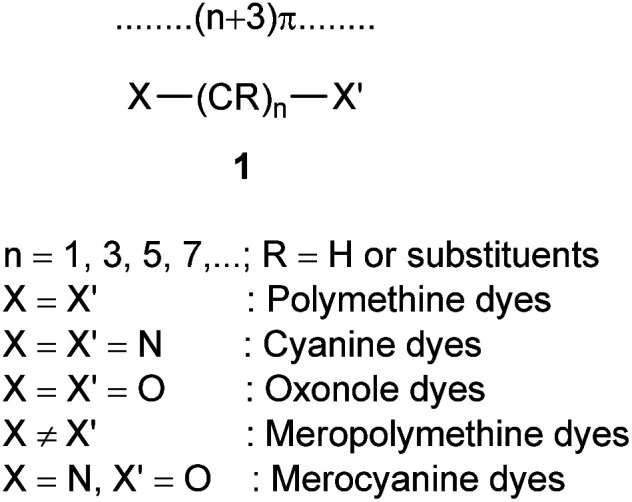


According to formula 1, cyanine dyes are a conjugate chain of carbon atoms located between two nitrogen centers, which this polymethine bridge links an electron donor group at one end and an electron acceptor group at the other. Conjugation between electron donor and acceptor group leads to delocalization of π electrons and hence positive charge over the two nitrogen atoms. The dye with 3 methine groups (*n* = 1 in formula 1) is classified as carbocyanine dyes or trimethine cyanine dyes. The wavelength of the cyanine dye absorption/emission depends on the nature of the end groups and the length of the polymethine chain. Monomethine and trimethine dyes are usually absorbed in the visible region (500–600 nm) of the electronic spectrum, and with each added methine unit (CH=CH) cause a bathochromic shift of about 100 nm in the electronic spectrum, resulting in absorption on the wavelength of 700–800 nm for penta- and heptamethine cyanines.^15–23^

**Scheme 1:**
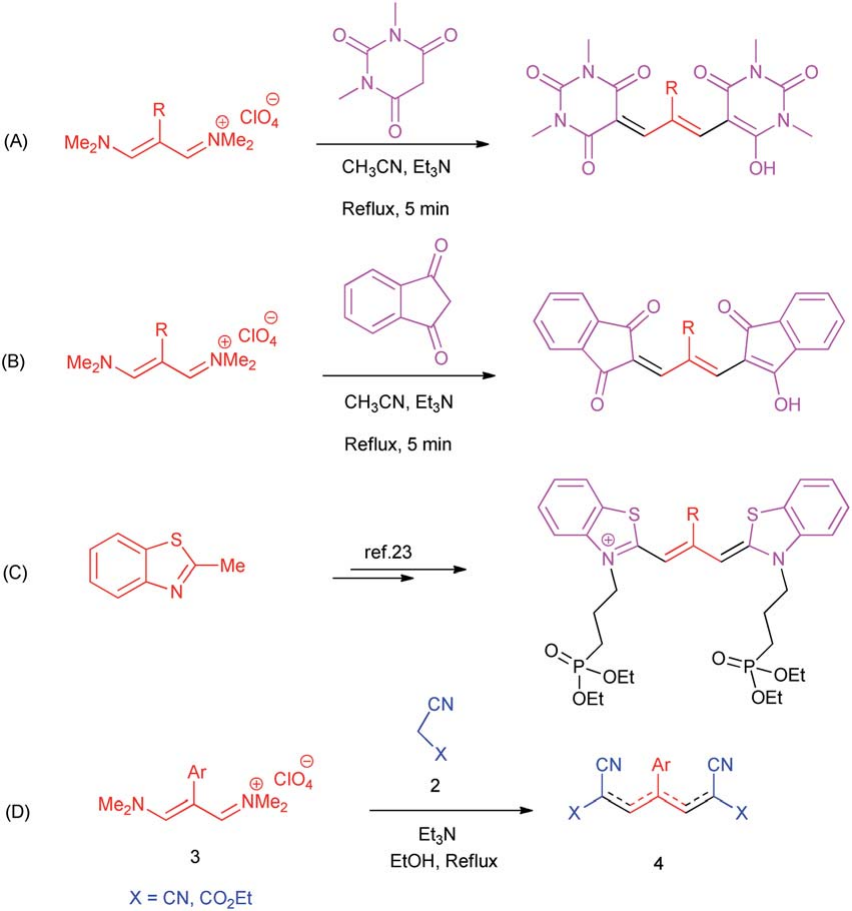
Representative condensation reactions towards cyanine dyes synthesis.

Due to the importance of cyanine dyes, researchers paid a lot of attention to provide different synthetic methods for the preparation of these structures. For example, in 2019, Naimi-Jamal obtained trimethine oxonol dyes from the reaction of 1,3-dimethylbarbituric acid with vinamidinium salts (Scheme 1A).^4^ They also produced other trimethine oxonol dyes from the same salts with 1,3-indandione (Scheme 1B).^24^ Mazières isolated trimethine benzothiazole cyanine dyes using the orthoester approach (Scheme 1C).^25^

In continuation of our interest in the development of trimethinium salts in organic synthesis,^26–30^ herein we report a simple and highly efficient synthetic procedure for the preparation of new zwitterionic pyridinium-cyanopropenides from the reaction of trimethinium salts and malononitrile derivatives in the presence of triethylamine in ethanol as a green solvent in excellent yields without any by-products (Scheme 1D).

## Results and discussion

The synthetic pathway for the synthesis of these new compounds is consisting of two steps. First, trimethinium salts 1 were prepared similar to the previous studies.^26–33^ Second, for the synthesis of novel zwitterionic pyridinium-cyanopropenides, trimethinium salts were isolated as perchlorate salts and reacted directly without additional purification were reacted with malononitrile derivatives in the presence of triethylamine in ethanol as a green solvent at reflux. Then, In order to optimize the reaction conditions for the synthesis of zwitterionic pyridinium-cyanopropenides derivatives, the model reaction between 1,3-bis(dimethylamino)-2-(isoquinolinium-2-yl)trimethinium bis(perchlorate) 3d and ethyl 2-cyanoacetate 2 was investigated in the presence of different bases and solvents. The results are summarized in Table 1. Different bases were evaluated in this study, such as NaH, Na_2_CO_3_, i-Pr_2_NEt, which lead to a low-yield product (Table 1, entries 1–3). Various solvents such as CH_3_CN, MeOH, and EtOH were studied, which CH_3_CN could not trigger this reaction (Table 1, entry 6), higher yield and shorter reaction time were obtained when the reaction was carried out in the presence of 1 mmol Et_3_N in ethanol as a solvent at reflux conditions (Table 1, entry 4). The control experiment confirmed that the reaction has not occurred under neutral and acid conditions (Table 1, entries 7 and 8).

**Table 1:** Optimization of the reaction conditions

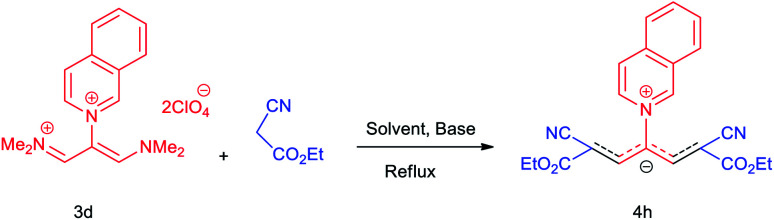				
Entry	Conditions	Solvent	Time (h)	Yield^a^ (%)
1	NaH	EtOH	5	40
2	Na_2_CO_3_	EtOH	5	40
3	i-Pr_2_NEt	EtOH	5	80
4	Et_3_N	EtOH	5	95
5	Et_3_N	MeOH	24	25
6	Et_3_N	CH_3_CN	24	—
7	—	EtOH	24	—
8	AcOH	EtOH	24	—

a Isolated yield.

Then, the scope and efficiency of the process were explored under optimized conditions. For this purpose, 2-substituted trimethinium salts 3a–d were condensed with malononitrile or ethyl 2-cyanoacetate in the presence of Et_3_N (1 mmol) to produce corresponding products 4. The obtained products 4a–h are betaine derived from nitrogen-heteroaryl trimethinium salts 3a–d. “Betaines are zwitterionic compounds with a full negative and a full positive charge in the same molecule, according to trimethylammonio acetate, Me_3_N^+^–CH_2_–CO_2_^−^.^33^ The results are shown in Table 2.

**Table 2:** Synthesis of product 4*via* the reaction of 2-substituted trimethinium salts 3 with malononitrile and ethyl cyanoacetate in the presence of Et_3_N in ethanol at reflux

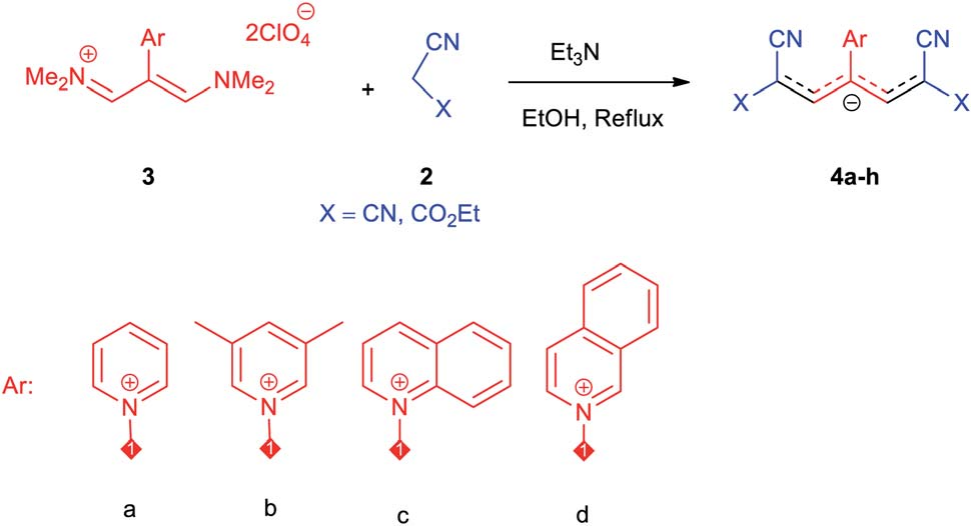				
Entry	Trimethinium salts	X	Product 3	Yield^a^ (%)
1	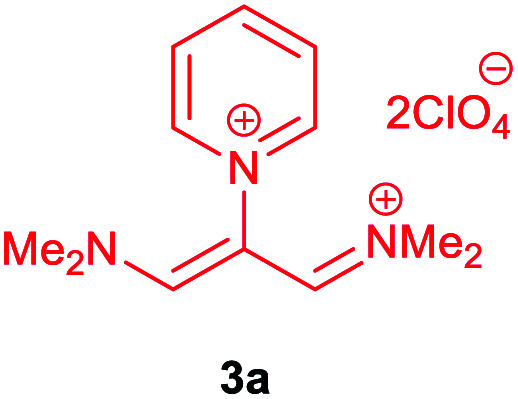	CN	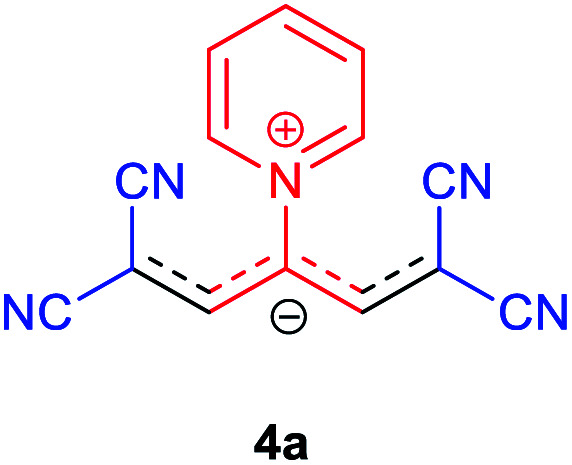	95
2	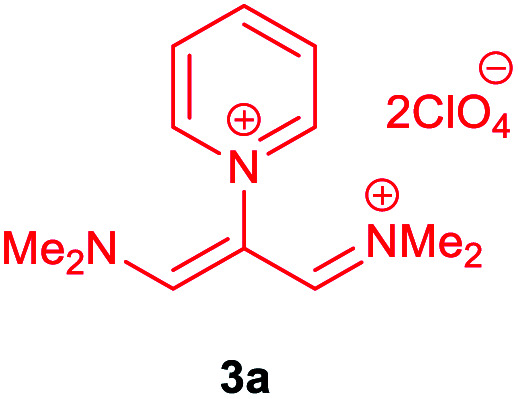	CO_2_Et	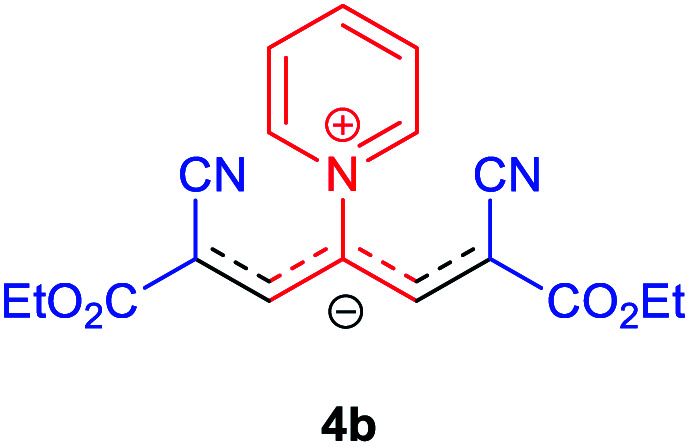	90
3	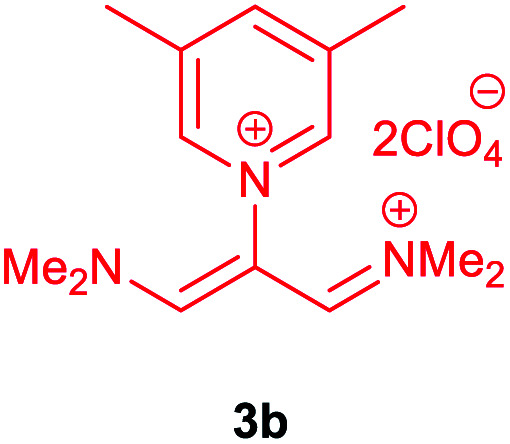	CN	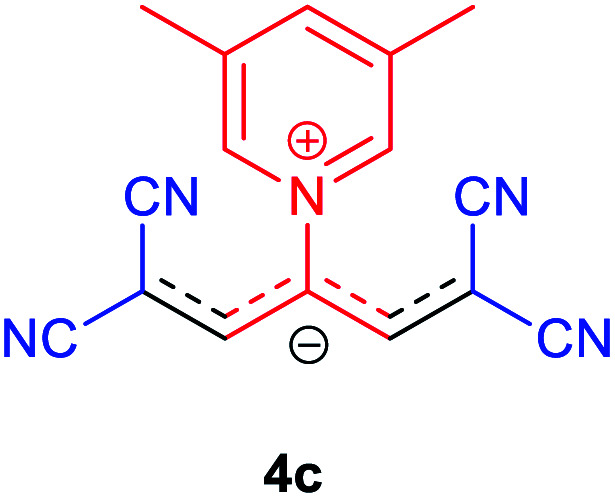	94
4	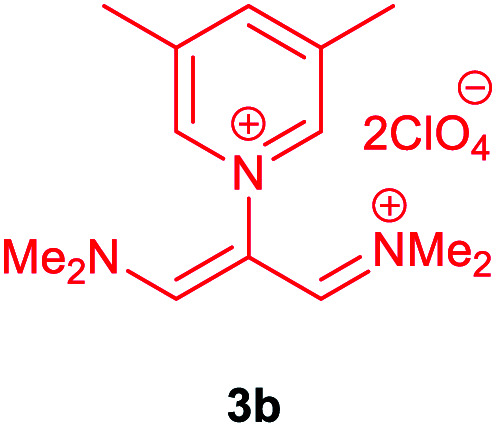	CO_2_Et	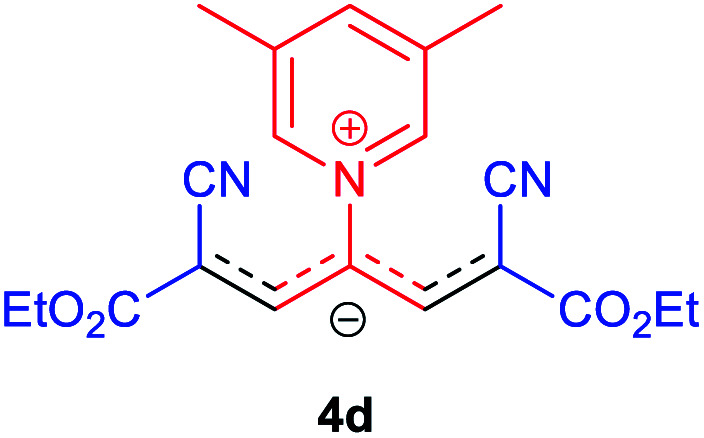	95
5	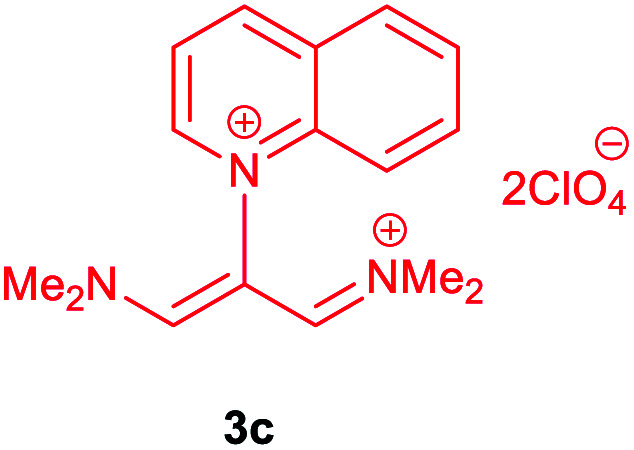	CN	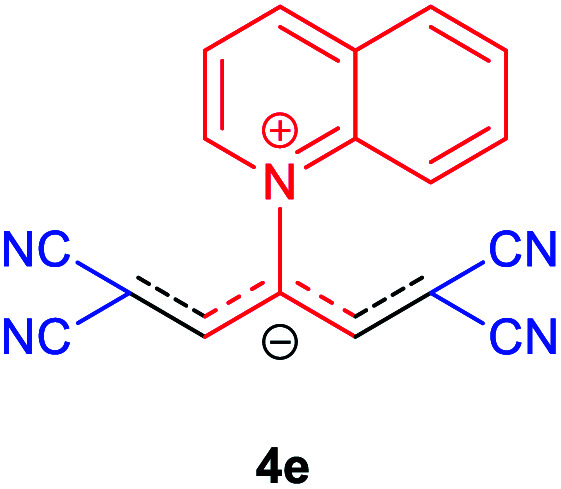	98
6	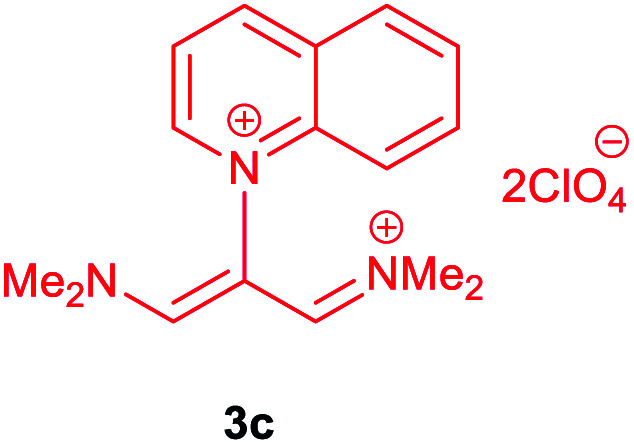	CO_2_Et	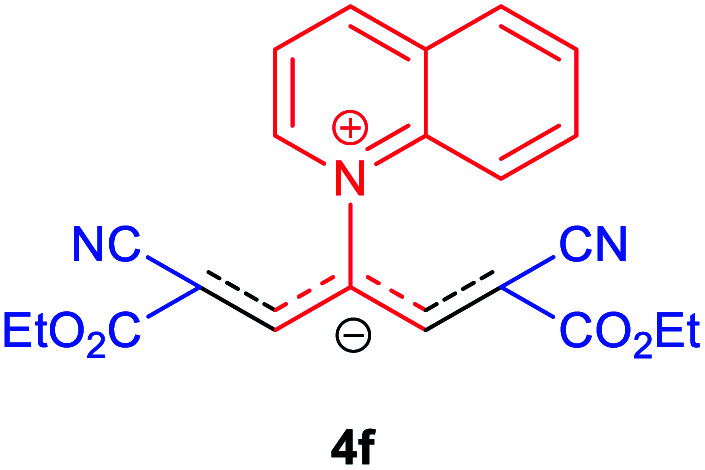	92
7	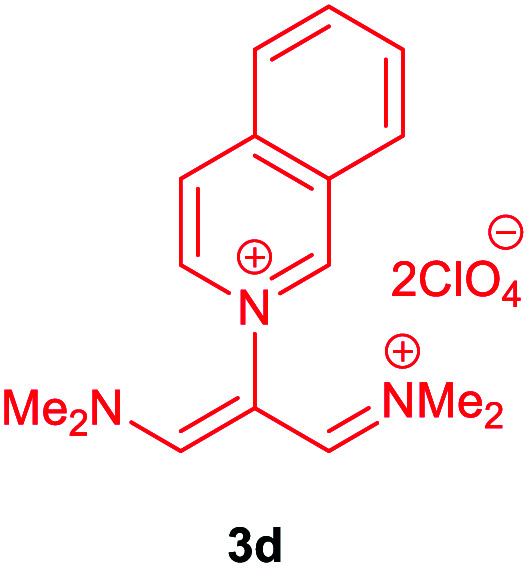	CN	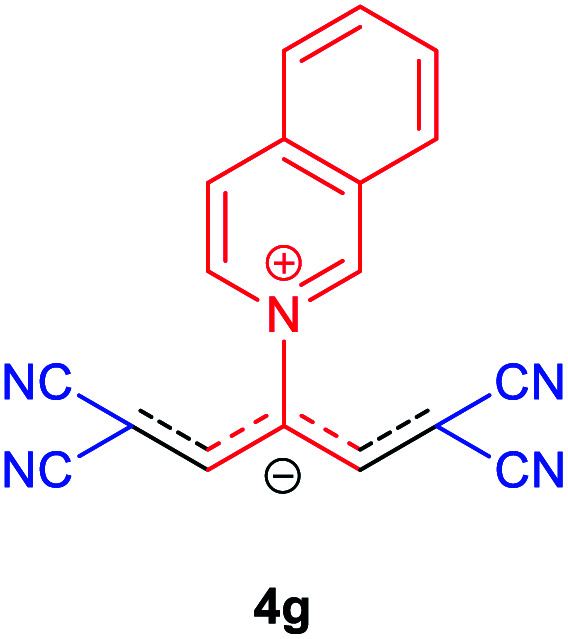	90
8	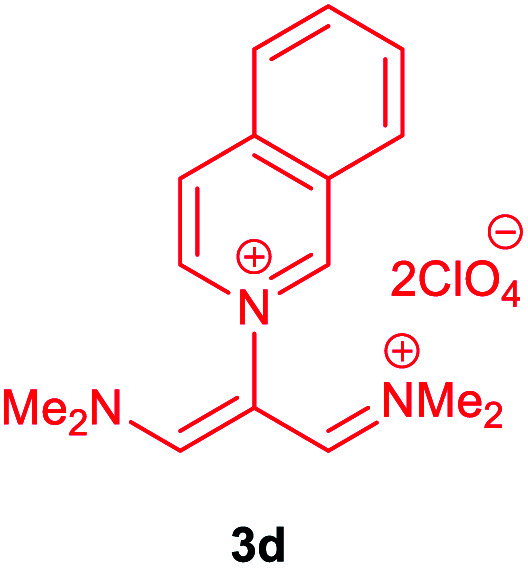	CO_2_Et	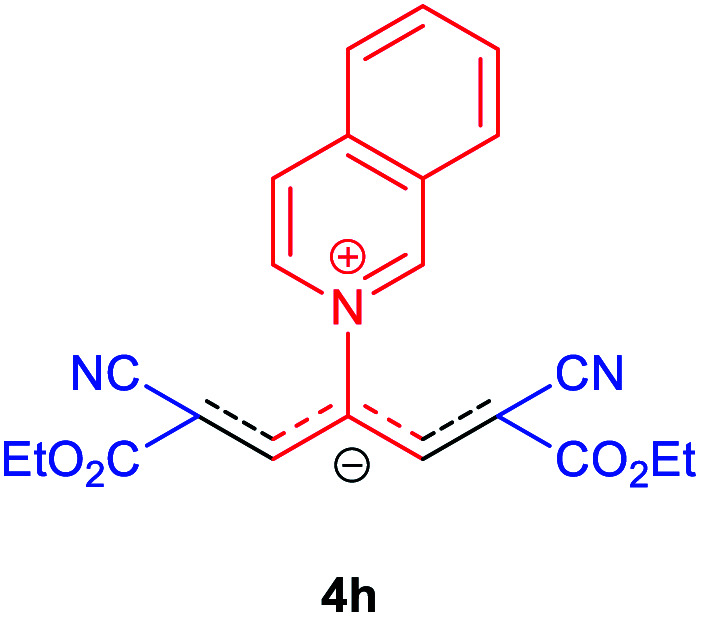	95

a Isolated yield.

The molecular structures of 4a–h were established by IR, UV/vis, mass, ^1^H and ^13^C NMR spectra as well as elemental analyses. The *λ*_max_ values of 4a–h were determined in DMSO in the spectral range of 200–400 nm.

**Scheme 2:**
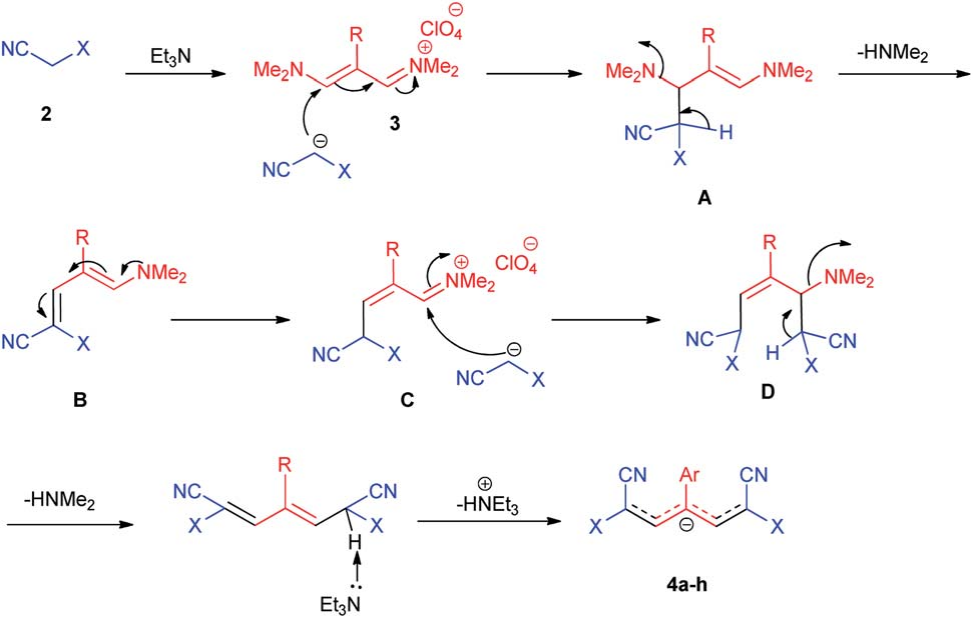
Proposed reaction mechanism for the synthesis of 4a–h.

The proposed reaction mechanism of the formation of 4a–h in the presence of Et_3_N is shown in Scheme 2. In the initial step, the methylene group in structure 2 is deprotonated in the presence of Et_3_N, followed by intermediate A is formed by the nucleophilic attack of the carbanion group in malono to trimethinium salt 3. Then, removal of dimethylamine occurs and intermediate B is formed. In the next step, the nucleophilic attack of the second molecule of the carbanion group in malono on the obtained iminium salt C produce intermediate D. Finally, with the loss of the second molecule of dimethylamine and acidic hydrogen by base, the desired betaine products are created.

## Experimental

All chemicals were purchased from Merck or Fluka chemical companies. The ^1^H NMR (300 MHz) and ^13^C NMR spectra (75 MHz) were run on a Bruker Avance 400. Tetramethylsilane (TMS) was used as the internal standard for the NMR analysis. IR spectra were recorded using an FTIR apparatus. Melting points were recorded on a Stuart Scientific Apparatus SMP3 (UK) in open capillary tubes. Elemental C, H and N analyses, were performed using a Costech CHNS–O elemental analyzer. UV/vis absorption spectra were recorded at room temperature in DMSO using a PerkinElmer Lambda 25 spectrophotometer.

### General procedure for the synthesis of pyridinium-cyanopropenides 4a–h

A solution of a 2-heteroraryl-substituted trimethinium salt 3a–d (1 mmol), malononitrile (2 mmol) or ethyl cyanoacetate (2 mmol) and triethylamine (1 mmol) in ethanol (10 mL) was heated under reflux for 5 h. Then, after cooling to room temperature, the precipitate formed was filtered off, washed with 2-propanol (3 × 3 mL), and dried under vacuum at 80 °C, to yield the desired betaine product 4a–h in excellent yields.

### 1,1,5,5-Tetracyano-3-(pyridinium-1-yl) penta propenides (4a)

Red powder, mp. > 260 °C, ^1^H NMR (DMSO-*d*_6_, 300 MHz), *δ* (ppm): 7.76 (s, 2H), 8.36 (t, *J* = 6.6 Hz, 2H), 8.81 (t, *J* = 7.6 Hz, 1H), 9.19 (d, *J* = 5.1 Hz, 2H). ^13^C NMR (DMSO-*d*_6_, 75 MHz), *δ* (ppm): 53.8, 114.4, 118.2, 120.9, 129.6, 148.9, 150.1, 150.2. Anal. calcd for C_14_H_7_N_5_: C, 68.57; H, 2.88; N, 28.56%. Found: C, 68.55; H, 2.85; N, 28.57%. *λ*_max_ (DMSO) = 457 nm.

### 2,6-Dicyano-1,7-diethoxy-1,7-dioxo-4-(pyridinium-1-yl) penta-propenides (4b)

Orange powder, mp. > 260 °C, ^1^H NMR (DMSO-*d*_6_, 300 MHz), *δ* (ppm): 1.20 (t, *J* = 6.9 Hz, 6H), 4.13 (q, *J* = 6.9 Hz, 4H), 8.01 (s, 2H), 8.30 (t, *J* = 6.6 Hz, 2H), 8.75 (t, *J* = 7.5 Hz, 1H), 9.12 (d, *J* = 5.4 Hz, 2H). ^13^C NMR (DMSO-*d*_6_, 75 MHz) *δ* (ppm): 14.8, 60.7, 76.5, 116.7, 119.0, 129.2, 148.0, 153.1, 154.0, 165.5. Anal. calcd for C_18_H_17_N_3_O_4_: C, 68.71; H, 5.05; N, 12.38%. Found: C, 68.70; H, 5.05; N, 12.37%. *λ*_max_ (DMSO) = 452 nm.

### 1,1,5,5-Tetracyano-3-(3,5-dimethylpyridinium-1-yl) penta-propenides (4c)

Violet powder, mp. > 260 °C, ^1^H NMR (DMSO-*d*_6_, 300 MHz), *δ* (ppm): 2.51 (s, 6H), 7.74 (s, 2H), 8.53 (s, 1H), 8.93 (s, 2H). ^13^C NMR (DMSO-*d*_6_, 75 MHz), *δ* (ppm): 18.2, 53.8, 114.5, 118.2, 120.9, 139.6, 146.2, 149.1, 149.9. Anal. calcd for C_16_H_11_N_5_: C, 70.32; H, 4.06; N, 25.63%. Found: C, 70.33; H, 4.08; N, 25.62%. *λ*_max_ (DMSO) = 455 nm.

### 2,6-Dicyano-1,7-diethoxy-1,7-dioxo-4-(3,5-dimethylpyridinium-1-yl)-penta-propenides (4d)

Red powder, mp. > 260 °C, ^1^H NMR (DMSO-*d*_6_, 300 MHz), *δ* (ppm): 1.20 (t, *J* = 3.4 Hz, 6H), 2.51 (s, 6H), 4.11–4.16 (m, 4H), 7.99 (d, *J* = 3.6 Hz, 2H), 8.46 (s, 1H), 8.85 (s, 2H). ^13^C NMR (DMSO-*d*_6_, 75 MHz), *δ* (ppm): 14.8, 18.2, 60.6, 76.6, 116.7, 119.0, 139.1, 146.3, 147.7, 148.2, 165.5. Anal. calcd for C_20_H_21_N_3_O_4_: C, 65.38; H, 5.76; N, 11.44%. Found: C, 65.75; H, 5.77; N, 11.43%. *λ*_max_ (DMSO) = 453 nm.

### 1-(1,1,5,5-Tetracyano-3-(quinolinium-1-yl)-penta-propenides) (4e)

Red powder, mp. > 260 °C, ^1^H NMR (DMSO-*d*_6_, 300 MHz), *δ* (ppm): 7.97 (s, 2H), 8.12 (t, *J* = 7.5 Hz, 1H), 8.20 (d, *J* = 8.7 Hz, 1H), 8.32–8.40 (m, 2H), 8.60 (d, *J* = 7.8 Hz, 1H), 9.54 (d, *J* = 8.1 Hz, 1H), 9.65 (dd, *J* = 1.2, 5.7 Hz, 1H). ^13^C NMR (DMSO-*d*_6_, 75 MHz), *δ* (ppm): 53.9, 114.5, 116.6, 118.1, 119.1, 123.0, 130.1, 131.2, 131.7, 138.1, 140.8, 151.0, 151.1, 153.9. Anal. calcd for C_18_H_9_N_5_: C, 73.21; H, 3.07; N, 23.72%. Found: C, 73.22; H, 3.05; N, 23.74%. *λ*_max_ (DMSO) = 450 nm.

### 2,6-Dicyano-1,7-diethoxy-1,7-dioxo-4-(quinolinium-1-yl) penta-propenides (4f)

Red powder, mp. > 260 °C, ^1^H NMR (DMSO-*d*_6_, 300 MHz), *δ* (ppm): 1.16 (t, *J* = 6.9 Hz, 6H), 4.10 (q, *J* = 6.9 Hz, 4H), 8.07–8.12 (m, 2H), 8.21–8.34 (m, 4H), 8.57 (d, *J* = 7.8 Hz, 1H), 9.47 (d, *J* = 8.1 Hz, 1H), 9.58 (d, *J* = 5.4 Hz, 1H). ^13^C NMR (DMSO-*d*_6_, 75 MHz), *δ* (ppm): 14,8, 60.7, 76.7, 114.6, 116.8, 119.4, 122.9, 130.2, 130.7 131.5, 137.4, 141.2, 148.5, 150.2, 153.7, 165.4. Anal. calcd for C_22_H_19_N_3_O_4_: C, 67.86; H, 4.92; N, 10.79%. Found: C, 67.85; H, 4.90; N, 10.80%. *λ*_max_ (DMSO) = 455 nm.

### 1,1,5,5-Tetracyano-3-(isoquinolinium-2-yl) penta-propenides (4g)

Red powder, mp. > 260 °C, ^1^H NMR (DMSO-*d*_6_, 300 MHz), *δ* (ppm): 7.86 (s, 2H), 8.10 (t, *J* = 7.5 Hz, 1H), 8.31 (t, *J* = 7.6 Hz, 1H), 8.41 (d, *J* = 8.1, 1H), 8.58 (d, *J* = 8.1 Hz, 1H), 8.78 (d, *J* = 6.6 Hz, 1H), 8.85 (dd, *J* = 0.9, 6.6 Hz, 1H), 10.28 (s, 1H). ^13^C NMR (DMSO-*d*_6_, 75 MHz), *δ* (ppm): 53.9, 114.7, 118.3, 121.1, 127.4, 127.9, 128.1, 131.6, 132.1, 138.7, 138.8, 139.7, 150.3, 155.1. *υ*_max_ (kBr): 2224, 1611, 1541, 1248, 1056 cm^−1^. Anal. calcd for C_18_H_9_N_5_: C, 73.21; H, 3.07; N, 23.72%. Found: C, 73.22; H, 3.05; N, 23.74%. *λ*_max_ (DMSO) = 450 nm.

### 2,6-Dicyano-1,7-diethoxy-4-(isoquinolinium-2-yl)-1,7-dioxo-penta-propenides (4h)

Red powder, mp. > 260 °C, ^1^H NMR (DMSO-*d*_6_, 300 MHz), *δ* (ppm): 1.18 (t, *J* = 6.9 Hz, 6H), 4.13 (q, *J* = 6.9 Hz, 4H), 8.06–8.15 (m, 3H), 8.29 (t, *J* = 7.2 Hz, 1H), 8.41 (d, *J* = 8.1 Hz, 1H), 8.57 (d, *J* = 8.1 Hz, 1H), 8.71–8.80 (m, 2H), 10.22 (s, 1H). ^13^C NMR (DMSO-*d*_6_, 75 MHz), *δ* (ppm): 14.8, 60.7, 76.7, 116.9, 119.2, 127.1, 127.8, 128.3, 131.4, 131.7, 138.1, 138.5, 140.1, 148.2, 154.9, 165.5. Anal. calcd for C_22_H_19_N_3_O_4_: C, 67.86; H, 4.92; N, 10.79%. Found: C, 67.85; H, 4.90; N, 10.80%. *λ*_max_ (DMSO) = 455 nm.

## Conclusions

In conclusion, we report on a highly efficient, one-pot method for the synthesis of new zwitterionic pyridinium-cyanopropenides by reaction of 2-heteroaryl-substituted trimethinium salts with malononitrile or ethyl cyanoacetate in ethanol solution under reflux.

A simple procedure with excellent yields, mild reaction conditions, easy purification of the products, and absence of by-products, are the main advantages of this method.

## Conflicts of interest

There are no conflicts to declare.

## Supplementary Material

RA-012-D2RA02465A-s001
