# Supplementary material for: An efficient route to the synthesis of novel zwitterionic pyridinium-cyanopropenides with 3-heteroaryl-substituted trimethinium salts
Source: RSC Adv. 2022 May 30;12(25):16229–34. doi: 10.1039/d2ra02465a (PMC9150544; doi:10.1039/d2ra02465a)
Supplement: RA-012-D2RA02465A-s001 [file RA-012-D2RA02465A-s001.pdf]

Electronic Supplementary Material (ESI) for RSC Advances.
This journal is © The Royal Society of Chemistry 2022

An efficient route to the synthesis of novel zwitterionic pyridinium-cyanopropenides with 3-heteroaryl-substituted trimethinium salts

Ziba Rafiee Samani,[a] Abdolmohammad Mehranpour*[a]

Department of Chemistry, Faculty of Science, Persian Gulf University, Bushehr 75169, Iran.
Email: ammehranpour@hotmail.com

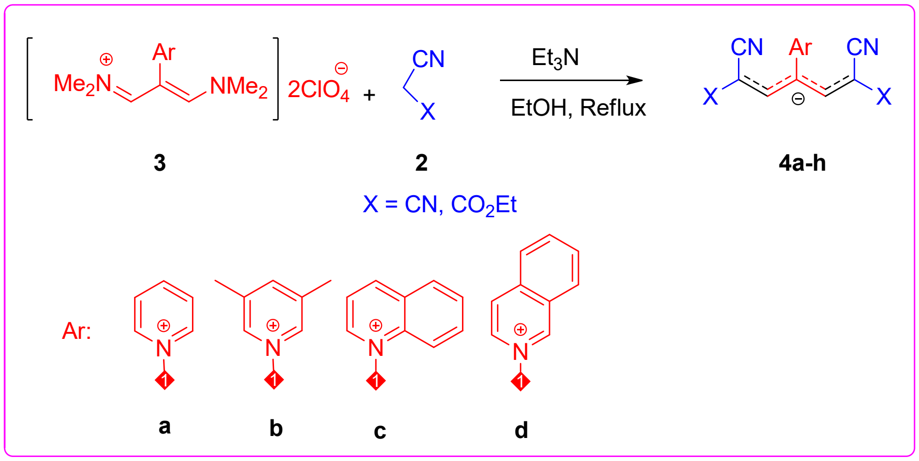

**4a**	**1,1,5,5-Tetracyano-3-(pyridinium-1-yl) penta propenides**	$C_{14}H_7N_5$
**4b**	**2,6-dicyano-1,7-diethoxy-1,7-dioxo-4-(pyridinium-1-yl) penta-propenides**	$C_{18}H_{17}N_3O_4$
**4c**	**1,1,5,5-tetracyano-3-(3,5-dimethylpyridinium-1-yl) penta-propenides**	$C_{16}H_{11}N_5$
**4d**	**2,6-dicyano-1,7-diethoxy-1,7-dioxo-4-(3,5-dimethylpyridinium-1-yl)-penta-propenides**	$C_{20}H_{21}N_3O_4$
**4e**	**1-(1,1,5,5-tetracyano-3-(quinolinium -1-yl) penta-propenides**	$C_{18}H_9N_5$
**4f**	**2,6-dicyano-1,7-diethoxy-1,7-dioxo-4-(quinolinium-1-yl) penta-propenides**	$C_{22}H_{19}N_3O_4$
**4g**	**1,1,5,5-tetracyano-3-(isoquinolinium-2-yl) penta-propenides**	$C_{18}H_9N_5$

4h	2,6-dicyano-1,7-diethoxy-4-(isoquinolinium-2-yl)-1,7-dioxo-penta-propenides	$C_{22}H_{19}N_3O_4$

IR, ^{1}H and ^{13}C NMR Spectra

1,1,5,5-Tetracyano-3-(pyridinium-1-yl)penta propenides (4a):

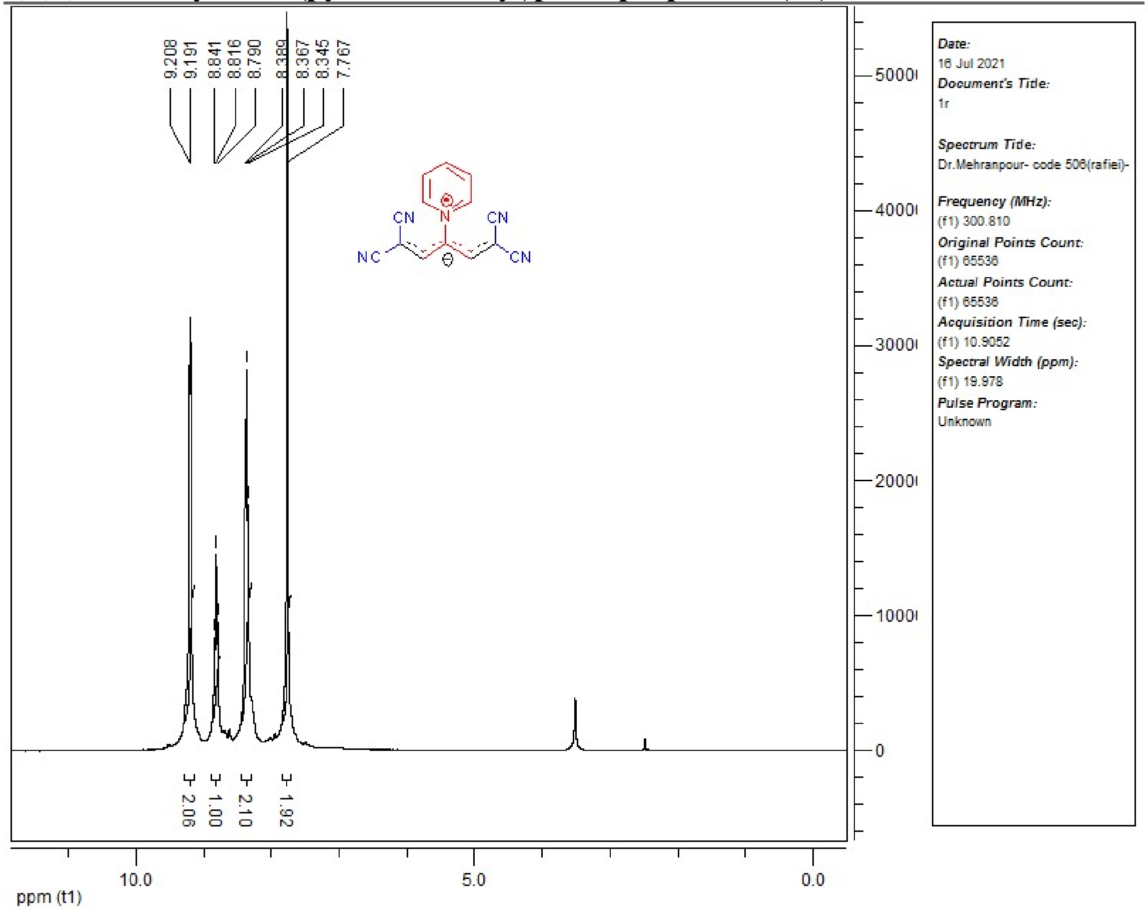

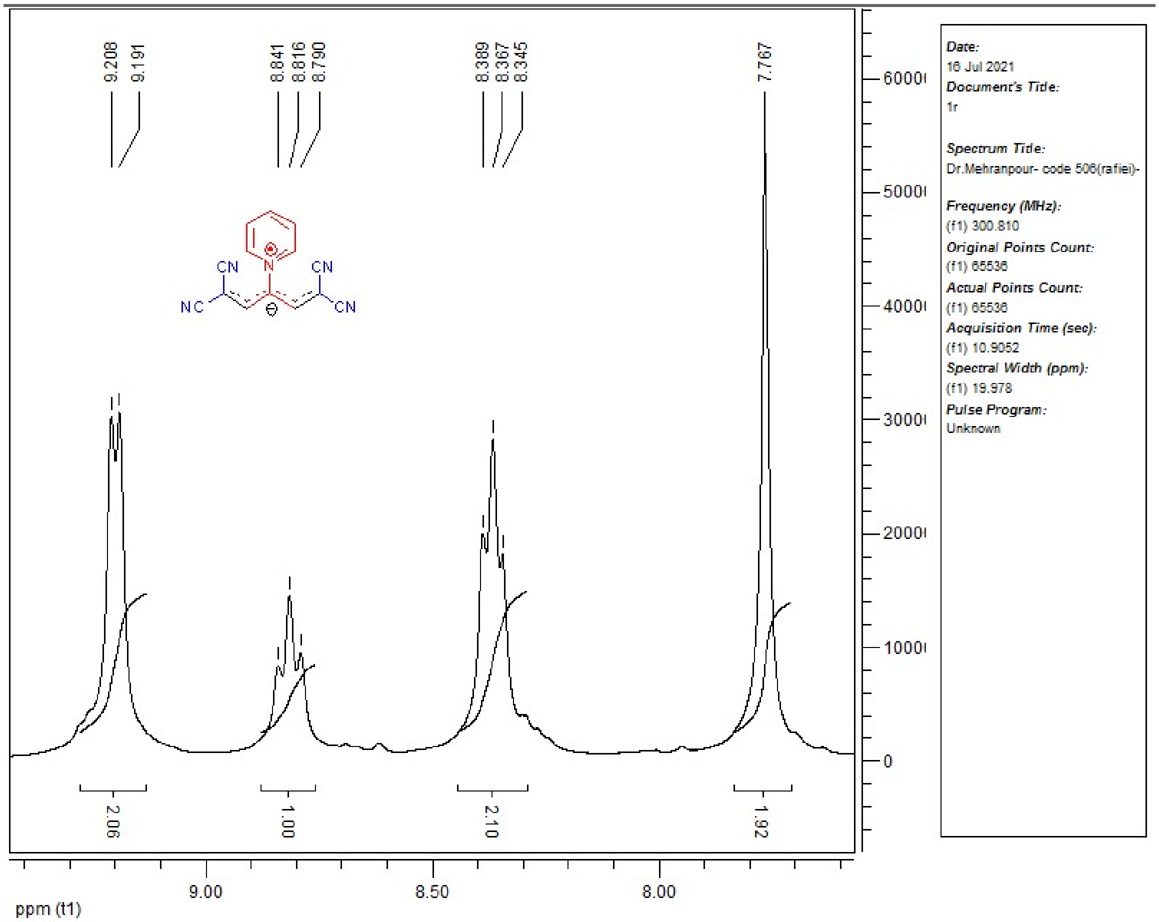

9.208
9.191
8.841
8.816
8.790
8.389
8.367
8.345
7.767
CN
CN
NC
CN
N
2.06
1.00
2.10
1.92
9.00
8.50
8.00
ppm (t1)
60000
50000
40000
30000
20000
10000
0
Date:
16 Jul 2021
Document's Title:
1r
Spectrum Title:
Dr.Mehranpour- code 506(rafiei)-
Frequency (MHz):
(f1) 300.810
Original Points Count:
(f1) 65536
Actual Points Count:
(f1) 65536
Acquisition Time (sec):
(f1) 10.9052
Spectral Width (ppm):
(f1) 19.978
Pulse Program:
Unknown

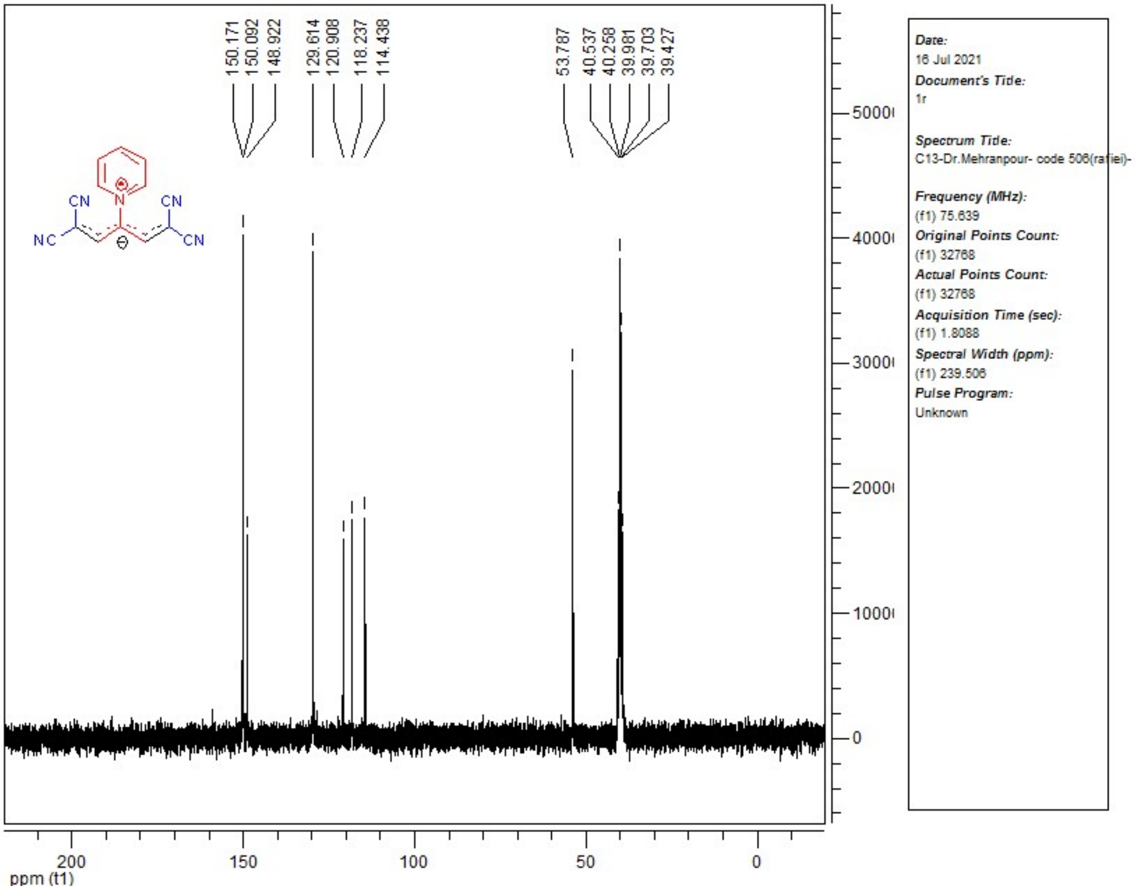

150.171
150.092
148.922
129.614
120.908
118.237
114.438
53.787
40.537
40.258
39.981
39.703
39.427
CN
NC
CN
CN
N
50000
40000
30000
20000
10000
0
200
150
100
50
0
ppm (t1)
Date:
16 Jul 2021
Document's Title:
1r
Spectrum Title:
C13-Dr.Mehranpour- code 506(rafiei)-
Frequency (MHz):
(f1) 75.639
Original Points Count:
(f1) 32768
Actual Points Count:
(f1) 32768
Acquisition Time (sec):
(f1) 1.8088
Spectral Width (ppm):
(f1) 239.506
Pulse Program:
Unknown

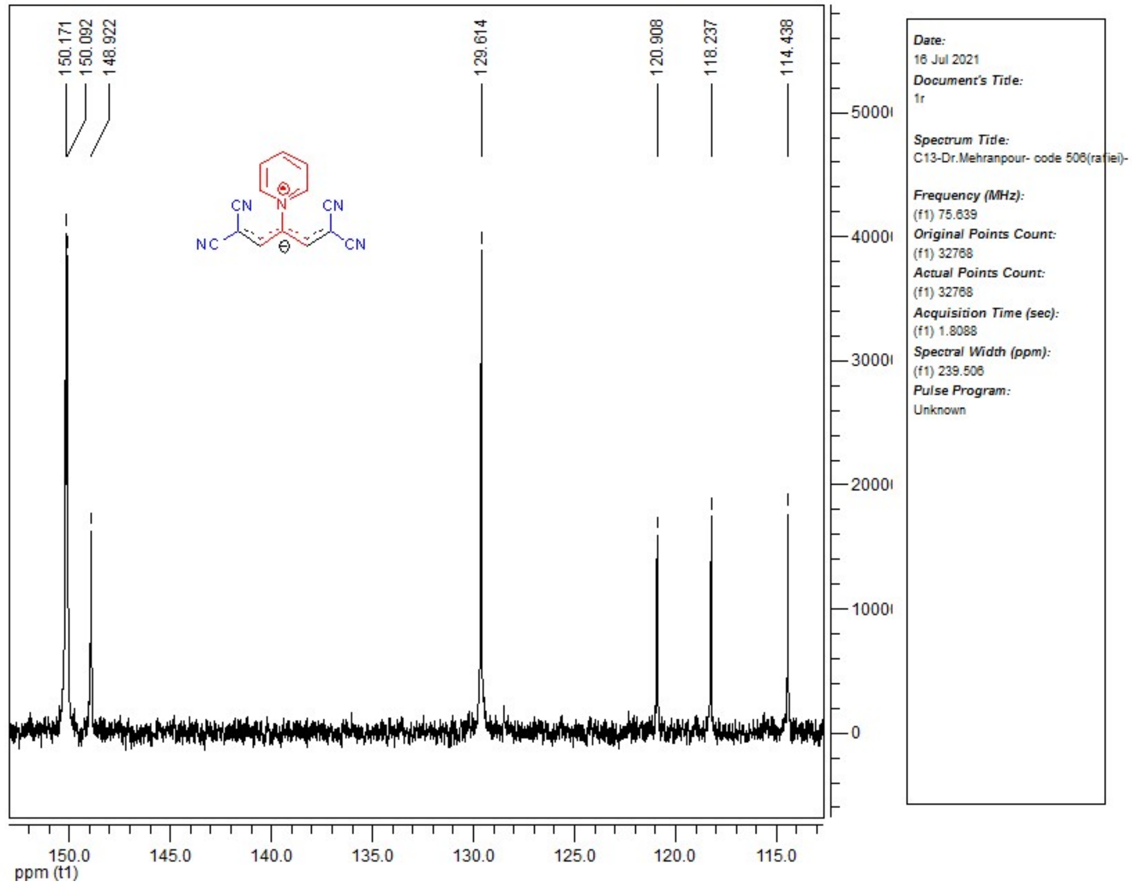

2,6-dicyano-1,7-diethoxy-1,7-dioxo-4-(pyridinium-1-yl)penta-propenides (4b):

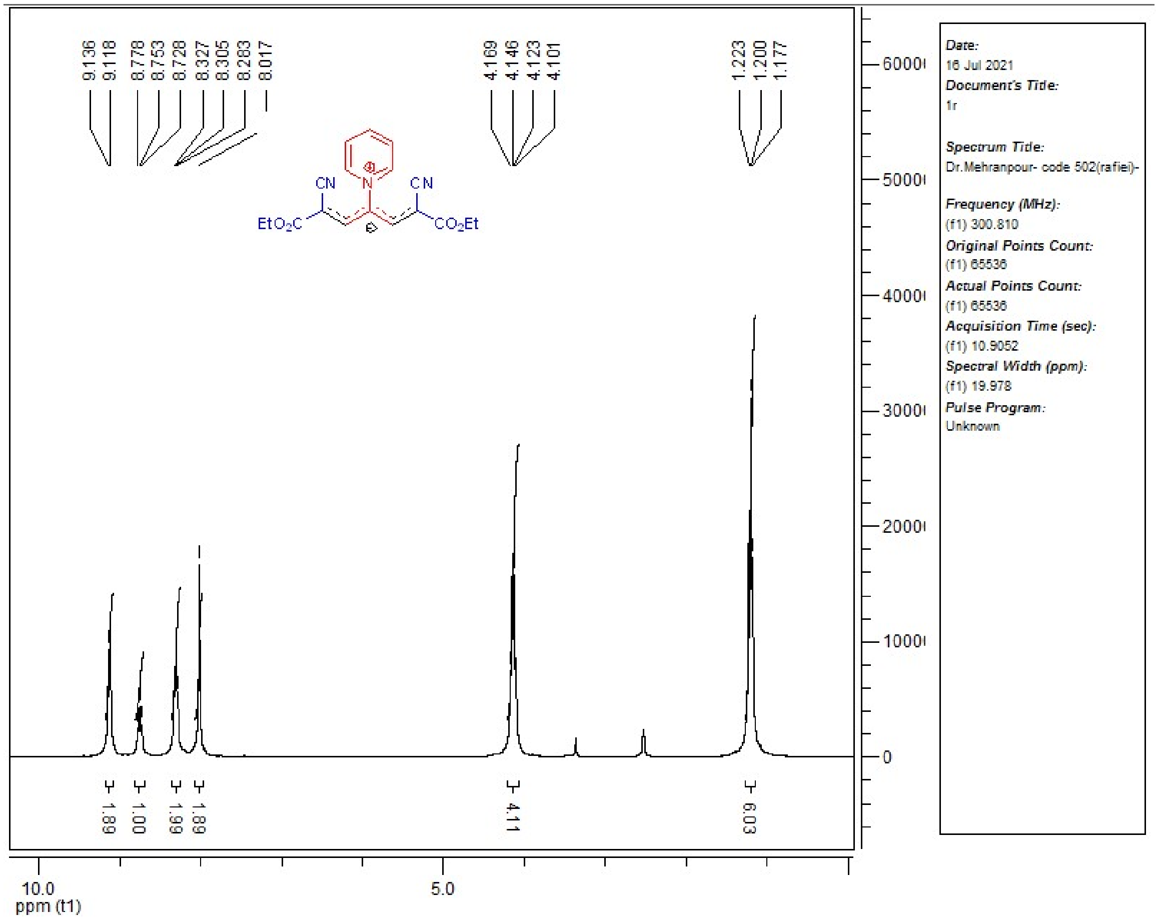
9.136
9.118
8.778
8.753
8.728
8.327
8.305
8.283
8.017
4.169
4.146
4.123
4.101
1.223
1.200
1.177
CN
CN
EtO2C
CO2Et
1.89
1.00
1.99
1.89
4.11
6.03
10.0
5.0
ppm (t1)
Date:
16 Jul 2021
Document's Title:
1r
Spectrum Title:
Dr.Mehranpour- code 502(rafiei)-
Frequency (MHz):
(f1) 300.810
Original Points Count:
(f1) 65536
Actual Points Count:
(f1) 65536
Acquisition Time (sec):
(f1) 10.9052
Spectral Width (ppm):
(f1) 19.978
Pulse Program:
Unknown

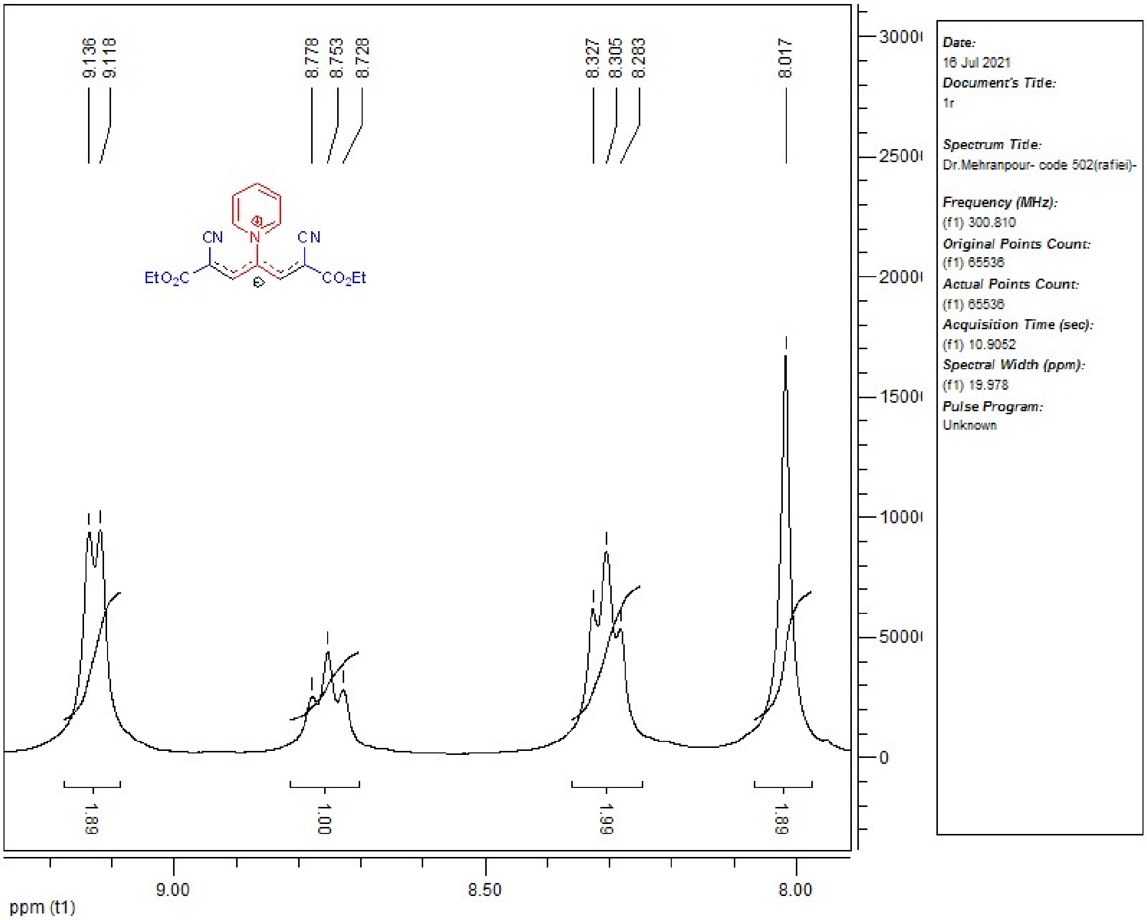
9.136
9.118
8.778
8.753
8.728
8.327
8.305
8.283
8.017
CN
CN
EtO2C
CO2Et
1.89
1.00
1.99
1.89
9.00
8.50
8.00
ppm (t1)
30000
25000
20000
15000
10000
5000
0
Date:
16 Jul 2021
Document's Title:
1r
Spectrum Title:
Dr.Mehranpour- code 502(rafiei)-
Frequency (MHz):
(f1) 300.810
Original Points Count:
(f1) 65536
Actual Points Count:
(f1) 65536
Acquisition Time (sec):
(f1) 10.9052
Spectral Width (ppm):
(f1) 19.978
Pulse Program:
Unknown

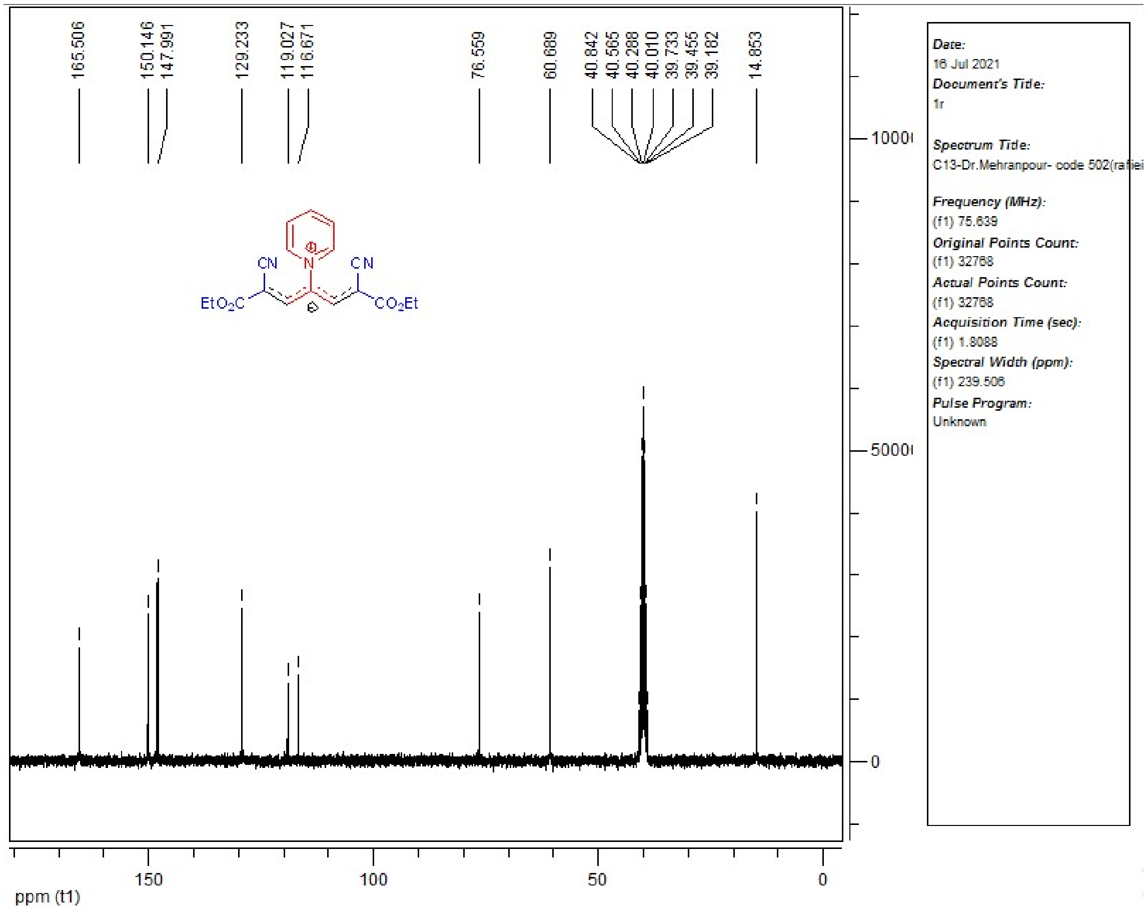

1,1,5,5-tetracyano-3-(3,5-dimethylpyridinium-1-yl)penta-propenides (4c):

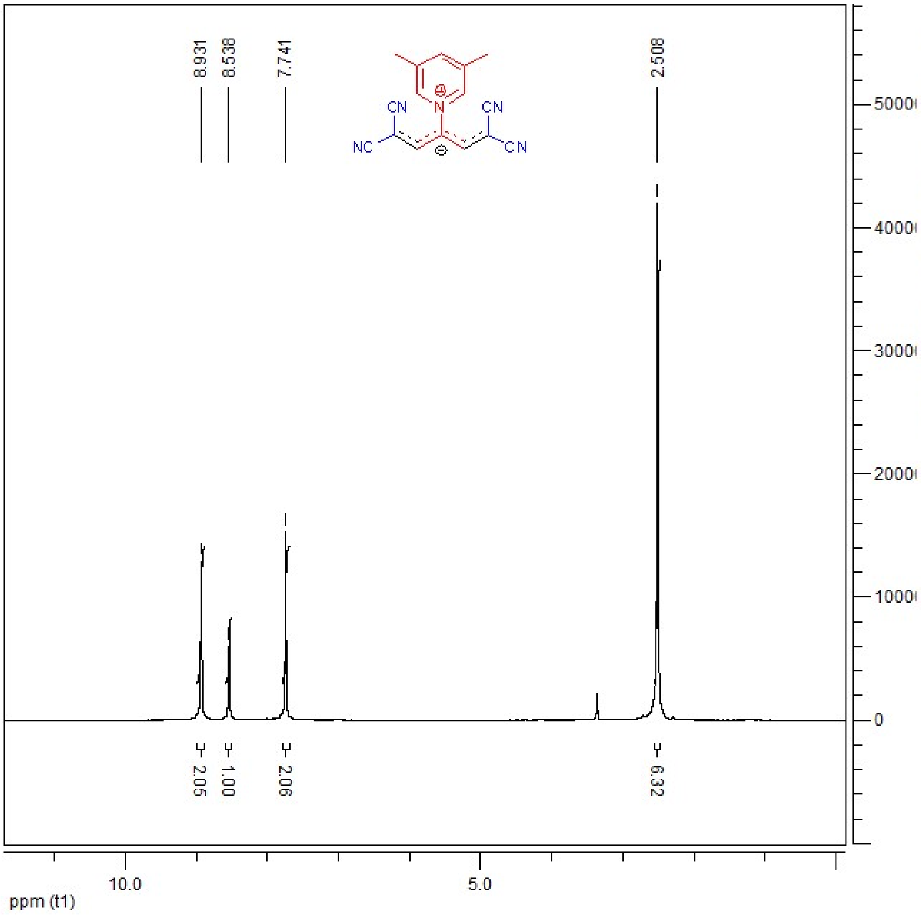

Date:
15 Jul 2021
Document's Title:
1r

Spectrum Title:
Dr.Mehranpour- code 501(rafiei)-

Frequency (MHz):
(f1) 300.810
Original Points Count:
(f1) 65536
Actual Points Count:
(f1) 65536
Acquisition Time (sec):
(f1) 10.9052
Spectral Width (ppm):
(f1) 19.978
Pulse Program:
Unknown

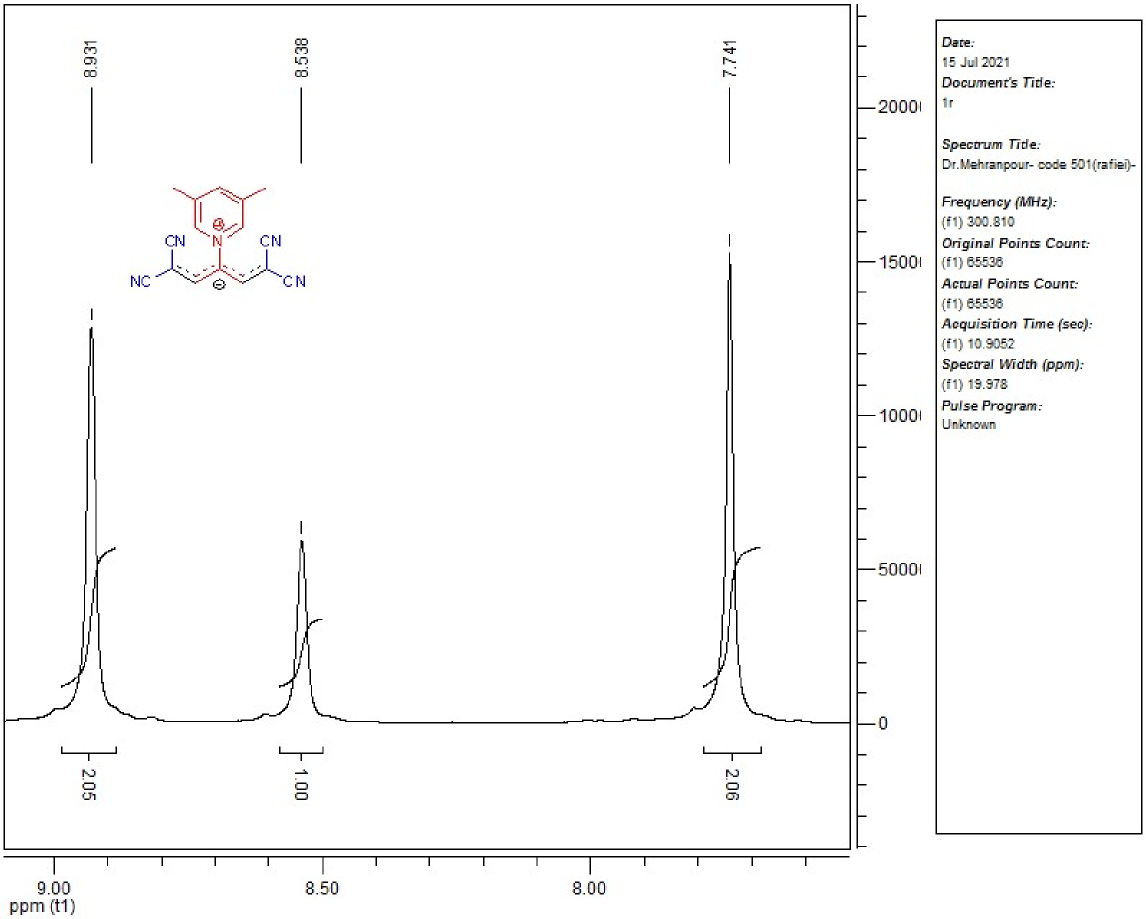
8.931
8.538
7.741
CN
NC
CN
CN
N
2.05
1.00
2.06
9.00
8.50
8.00
ppm (t1)
2000
1500
1000
5000
0
Date:
15 Jul 2021
Document's Title:
1r
Spectrum Title:
Dr.Mehranpour- code 501(rafiei)-
Frequency (MHz):
(f1) 300.810
Original Points Count:
(f1) 65536
Actual Points Count:
(f1) 65536
Acquisition Time (sec):
(f1) 10.9052
Spectral Width (ppm):
(f1) 19.978
Pulse Program:
Unknown

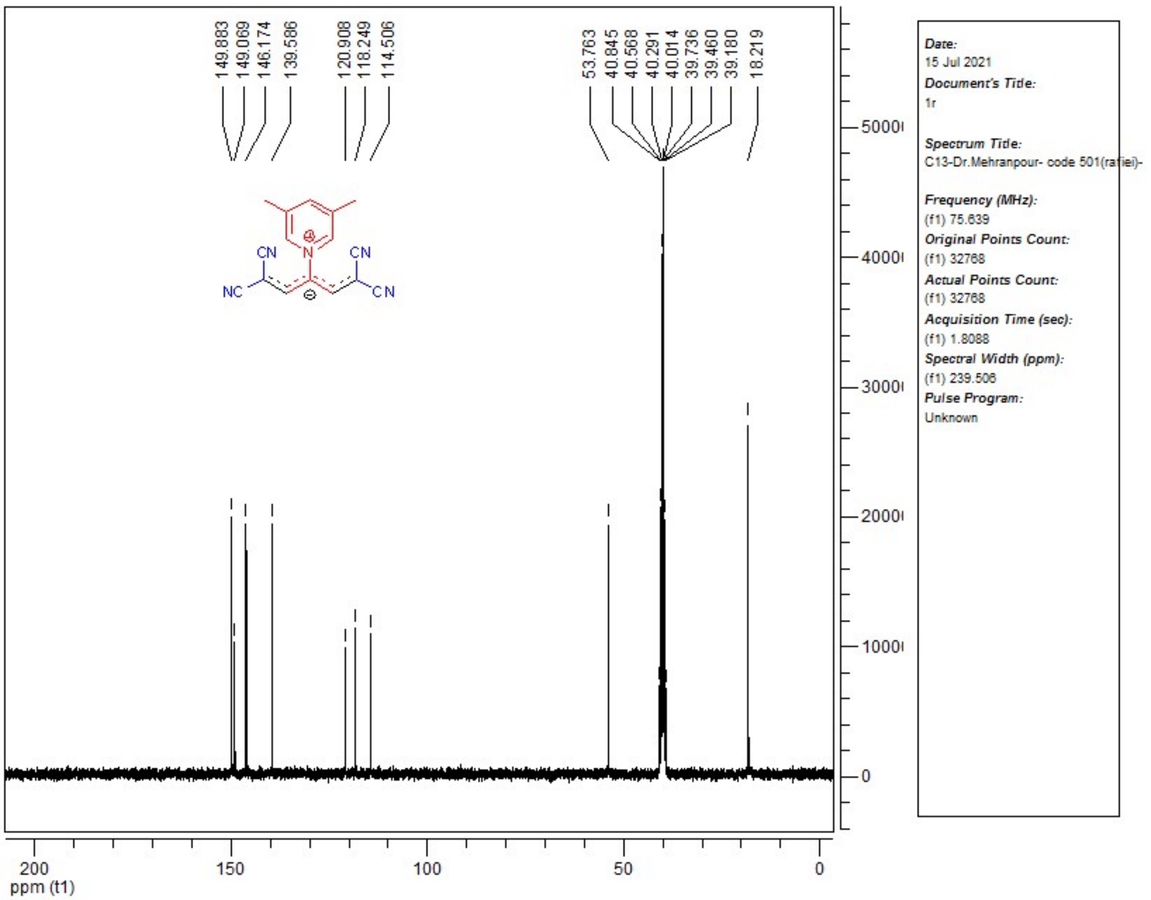
149.883
149.069
146.174
139.586
120.908
118.249
114.506
53.763
40.845
40.568
40.291
40.014
39.736
39.460
39.180
18.219
CN
NC
CN
CN
N
50000
40000
30000
20000
10000
0
200
150
100
50
0
ppm (t1)
Date:
15 Jul 2021
Document's Title:
1r
Spectrum Title:
C13-Dr.Mehranpour- code 501(rafiei)-
Frequency (MHz):
(f1) 75.639
Original Points Count:
(f1) 32768
Actual Points Count:
(f1) 32768
Acquisition Time (sec):
(f1) 1.8088
Spectral Width (ppm):
(f1) 239.506
Pulse Program:
Unknown

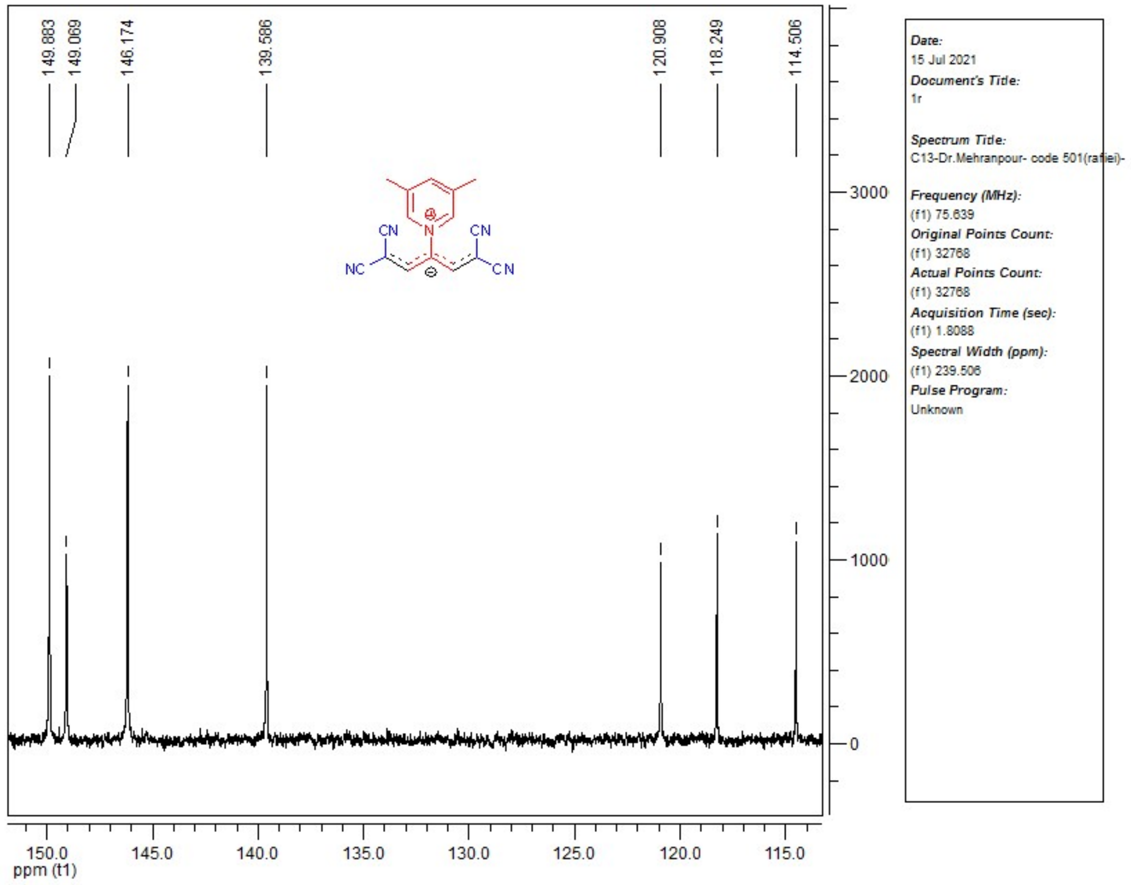

2,6-dicyano-1,7-diethoxy-1,7-dioxo-4-(3,5-dimethylpyridinium-1-yl)-penta-propenides (4d):

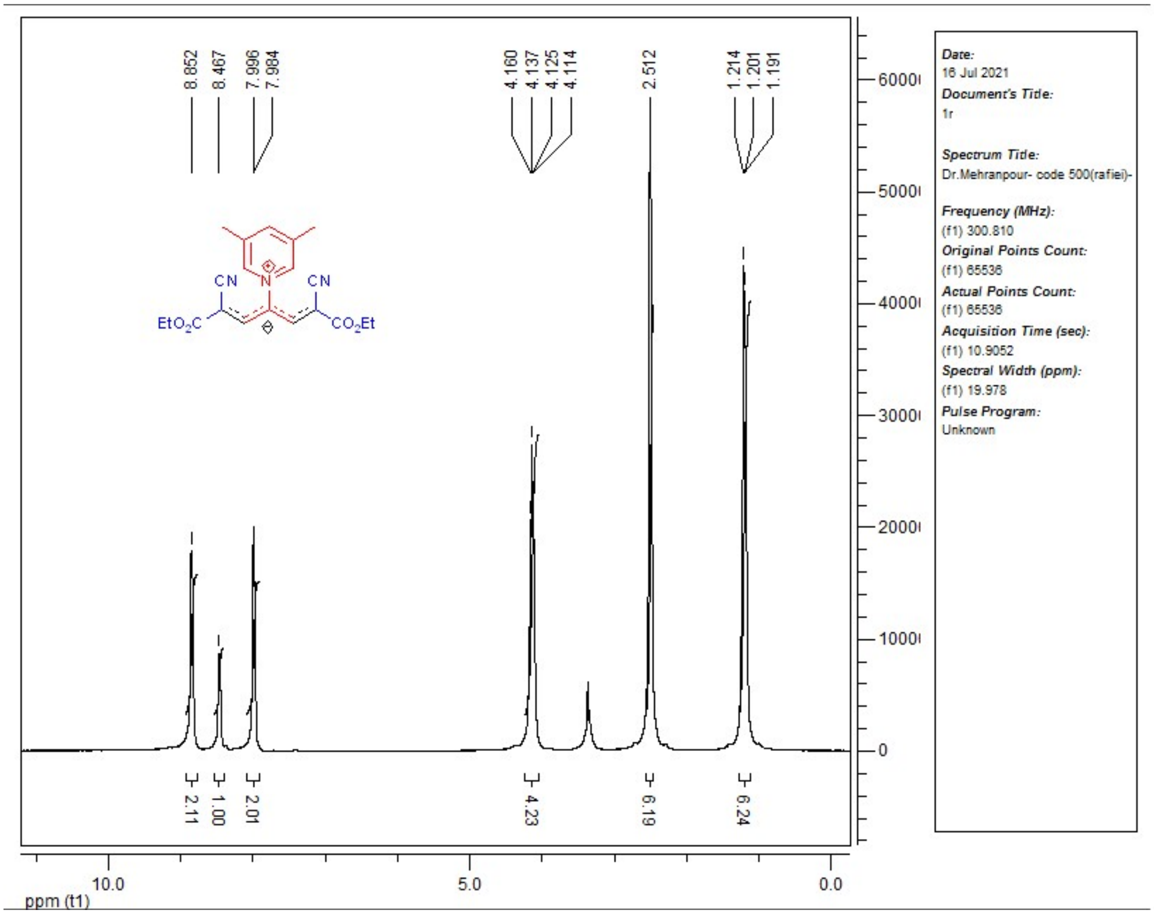

8.852
8.467
7.996
7.984
4.160
4.137
4.125
4.114
2.512
1.214
1.201
1.191
CN
CN
EtO2C
CO2Et
2.11
1.00
2.01
4.23
6.19
6.24
60000
50000
40000
30000
20000
10000
0
10.0
5.0
0.0
ppm (t1)
Date:
16 Jul 2021
Document's Title:
1r
Spectrum Title:
Dr.Mehranpour- code 500(rafiei)-
Frequency (MHz):
(f1) 300.810
Original Points Count:
(f1) 65536
Actual Points Count:
(f1) 65536
Acquisition Time (sec):
(f1) 10.9052
Spectral Width (ppm):
(f1) 19.978
Pulse Program:
Unknown

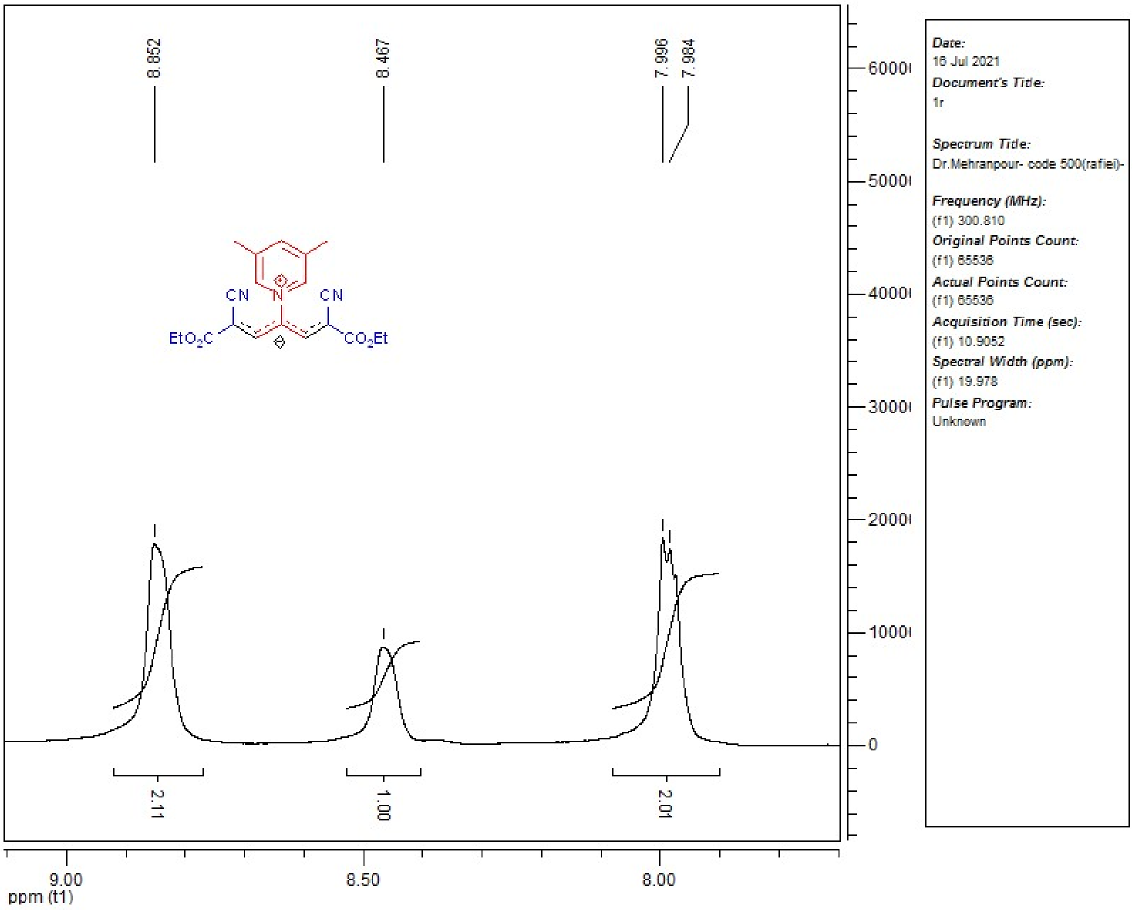
8.852
8.467
7.996
7.984
CN
CN
EtO2C
CO2Et
2.11
1.00
2.01
9.00
8.50
8.00
ppm (t1)
60000
50000
40000
30000
20000
10000
0
Date:
16 Jul 2021
Document's Title:
1r
Spectrum Title:
Dr.Mehranpour- code 500(rafiei)-
Frequency (MHz):
(f1) 300.810
Original Points Count:
(f1) 65536
Actual Points Count:
(f1) 65536
Acquisition Time (sec):
(f1) 10.9052
Spectral Width (ppm):
(f1) 19.978
Pulse Program:
Unknown

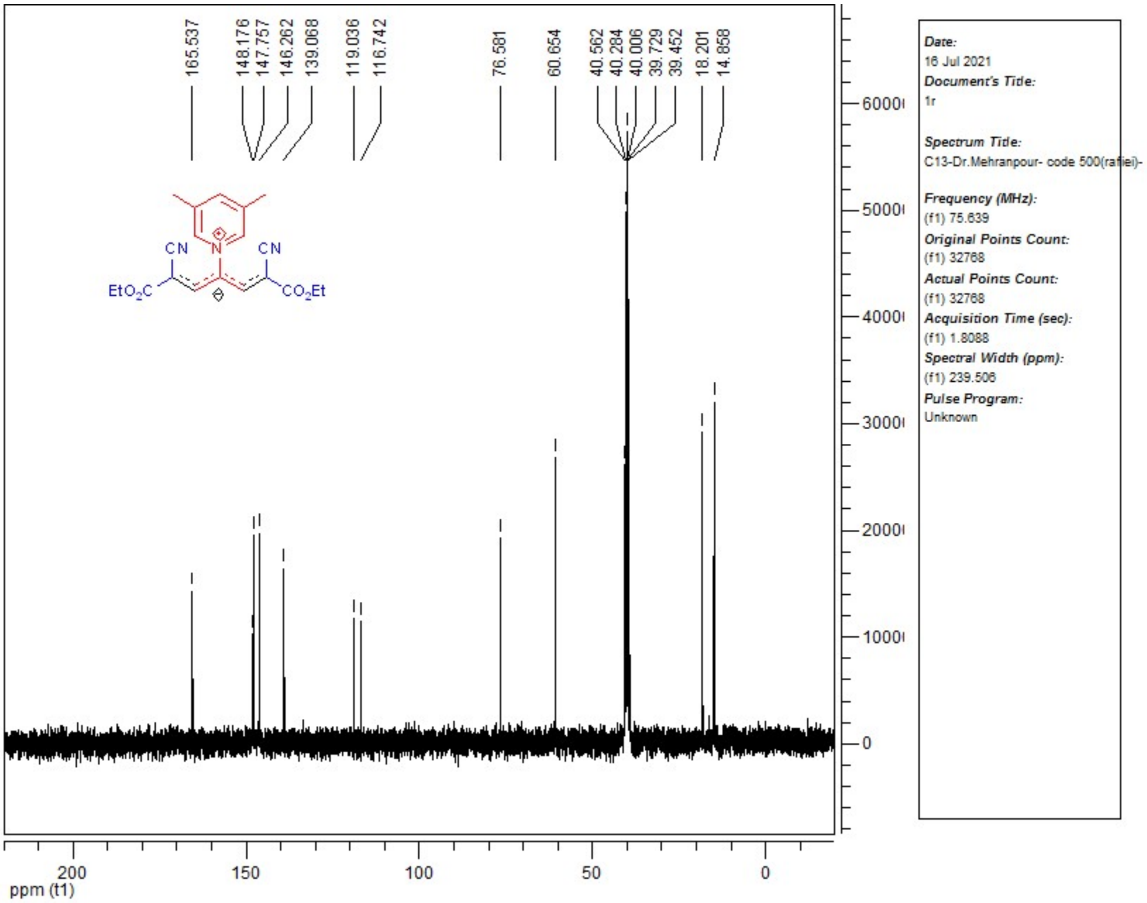
165.537
148.176
147.757
146.262
139.068
119.036
116.742
76.581
60.654
40.562
40.284
40.006
39.729
39.452
18.201
14.858
CN
CN
EtO2C
CO2Et
60000
50000
40000
30000
20000
10000
0
200
150
100
50
0
ppm (t1)
Date:
16 Jul 2021
Document's Title:
1r
Spectrum Title:
C13-Dr.Mehranpour- code 500(rafiei)-
Frequency (MHz):
(f1) 75.639
Original Points Count:
(f1) 32768
Actual Points Count:
(f1) 32768
Acquisition Time (sec):
(f1) 1.8088
Spectral Width (ppm):
(f1) 239.506
Pulse Program:
Unknown

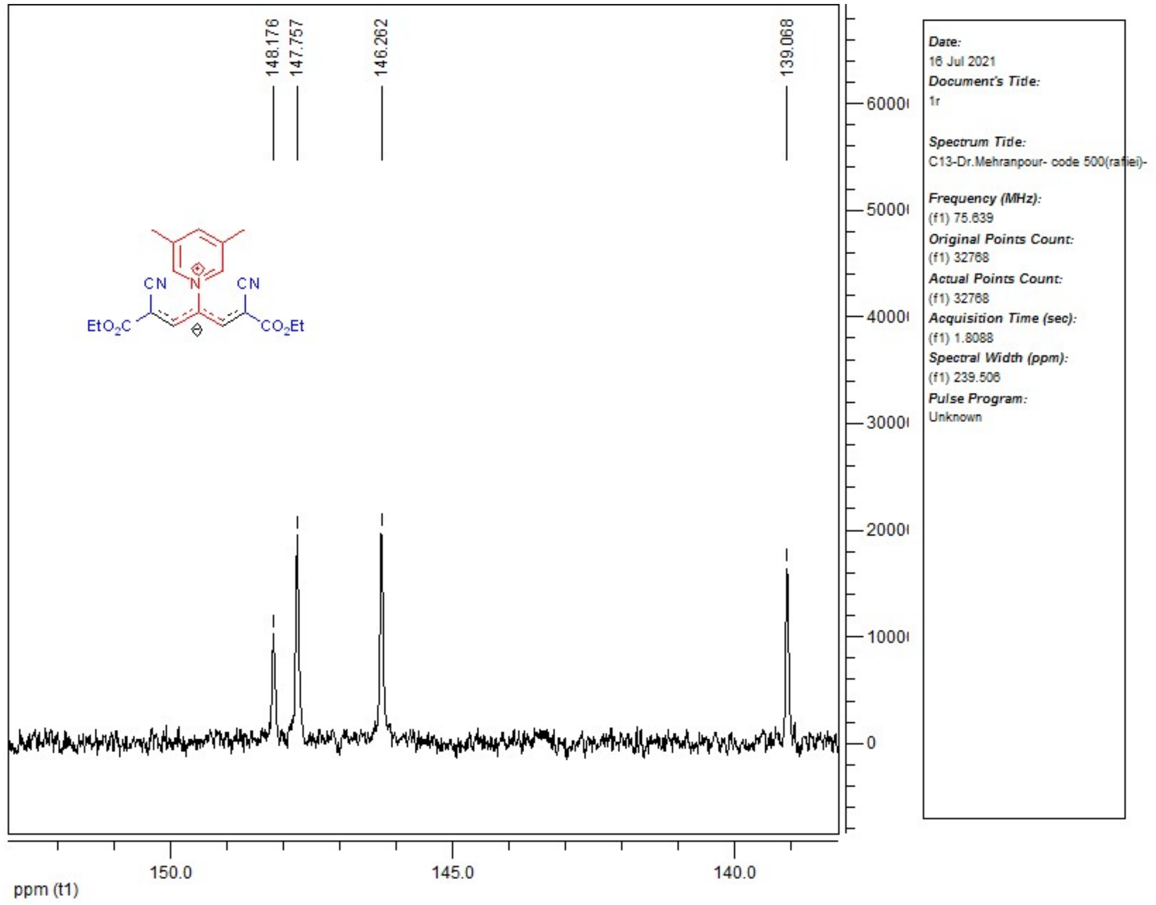

1-(1,1,5,5-tetracyano-3-(quinolinium -1-yl) penta-propenides (4e):

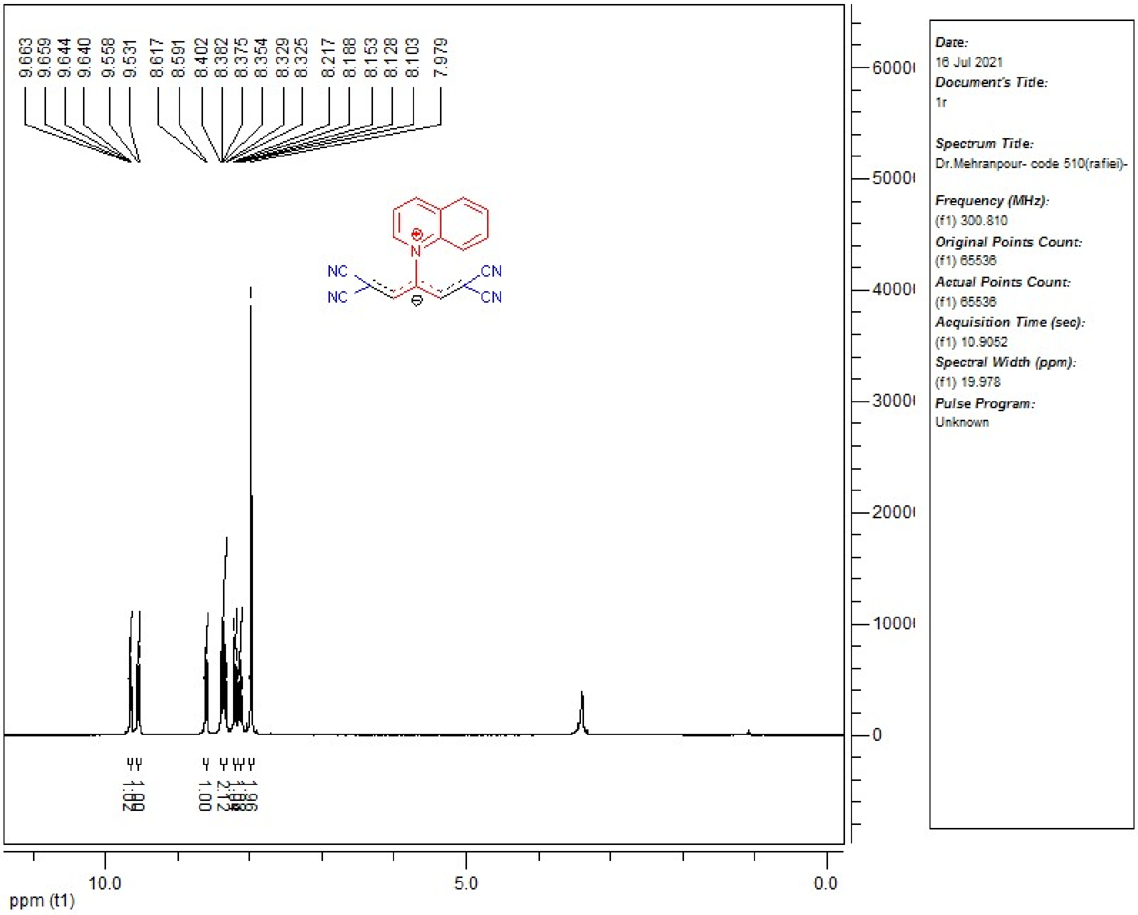
9.663
9.659
9.644
9.640
9.558
9.531
8.617
8.591
8.402
8.382
8.375
8.354
8.329
8.325
8.217
8.188
8.153
8.128
8.103
7.979
NC
NC
CN
CN
N
60000
50000
40000
30000
20000
10000
0
1.00
1.00
2.12
1.96
10.0
5.0
0.0
ppm (t1)
Date:
16 Jul 2021
Document's Title:
1r
Spectrum Title:
Dr.Mehranpour- code 510(rafiei)-
Frequency (MHz):
(f1) 300.810
Original Points Count:
(f1) 65536
Actual Points Count:
(f1) 65536
Acquisition Time (sec):
(f1) 10.9052
Spectral Width (ppm):
(f1) 19.978
Pulse Program:
Unknown

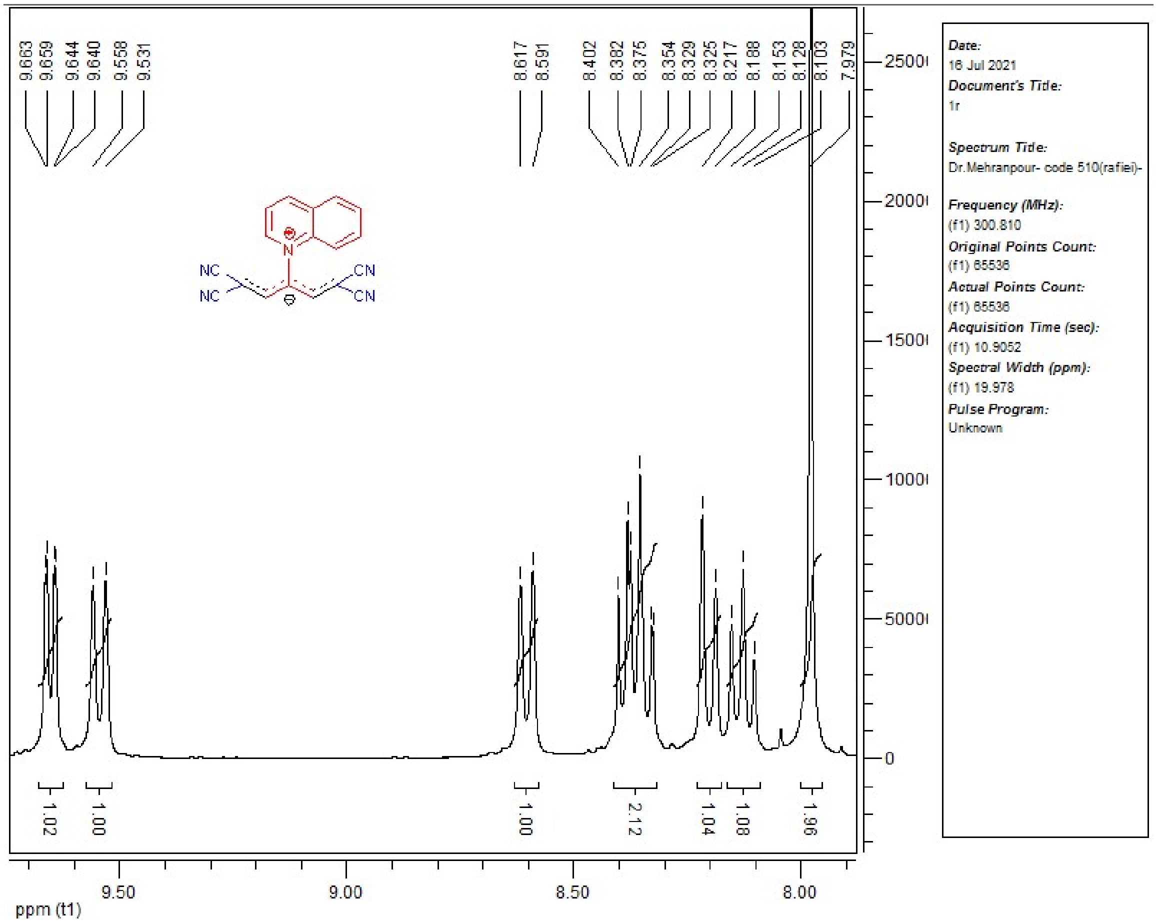
9.663
9.659
9.644
9.640
9.558
9.531
8.617
8.591
8.402
8.382
8.375
8.354
8.329
8.325
8.217
8.188
8.153
8.128
8.103
7.979
NC
NC
CN
CN
N
1.02
1.00
1.00
2.12
1.04
1.08
1.96
9.50
9.00
8.50
8.00
ppm (t1)
25000
20000
15000
10000
5000
0
Date:
16 Jul 2021
Document's Title:
1r
Spectrum Title:
Dr.Mehranpour- code 510(rafiei)-
Frequency (MHz):
(f1) 300.810
Original Points Count:
(f1) 65536
Actual Points Count:
(f1) 65536
Acquisition Time (sec):
(f1) 10.9052
Spectral Width (ppm):
(f1) 19.978
Pulse Program:
Unknown

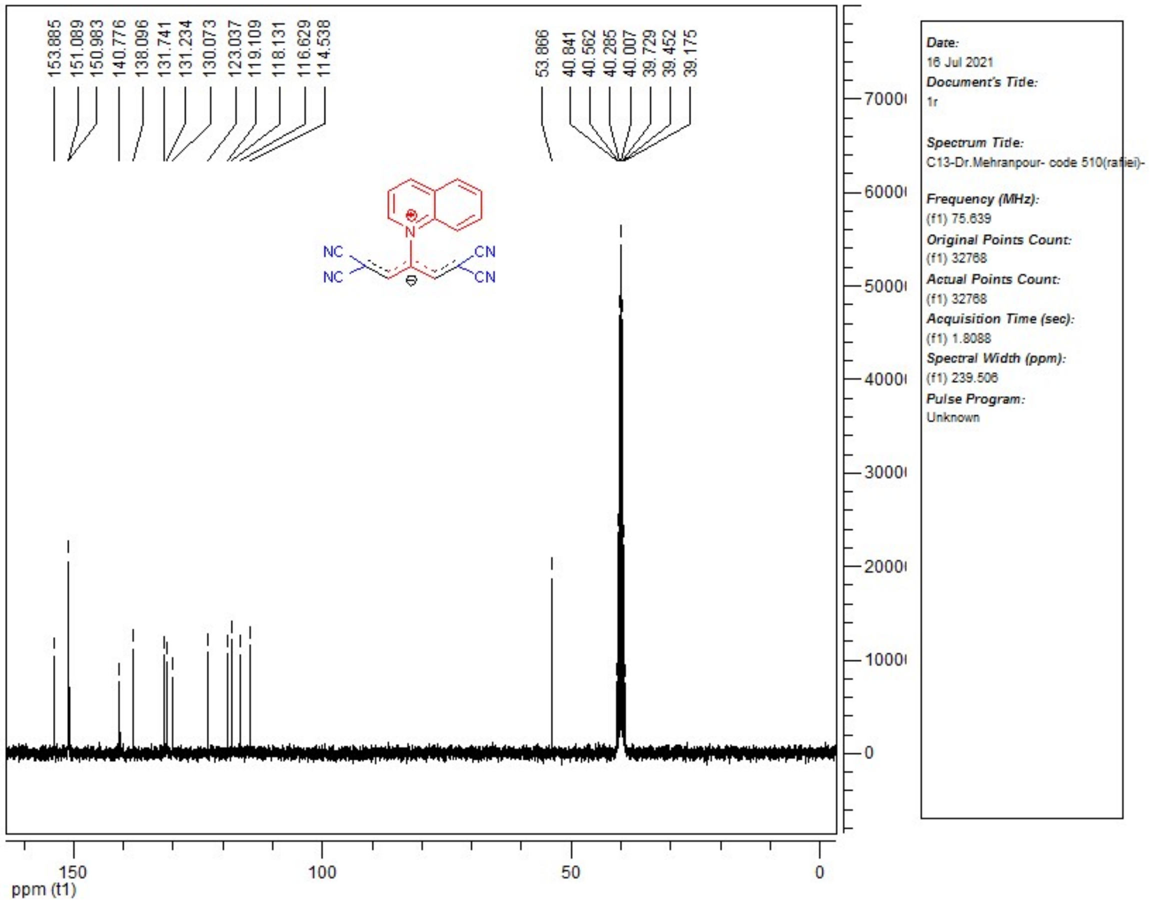

2,6-dicyano-1,7-diethoxy-1,7-dioxo-4-(quinolinium-1-yl)penta-propenides (4f):

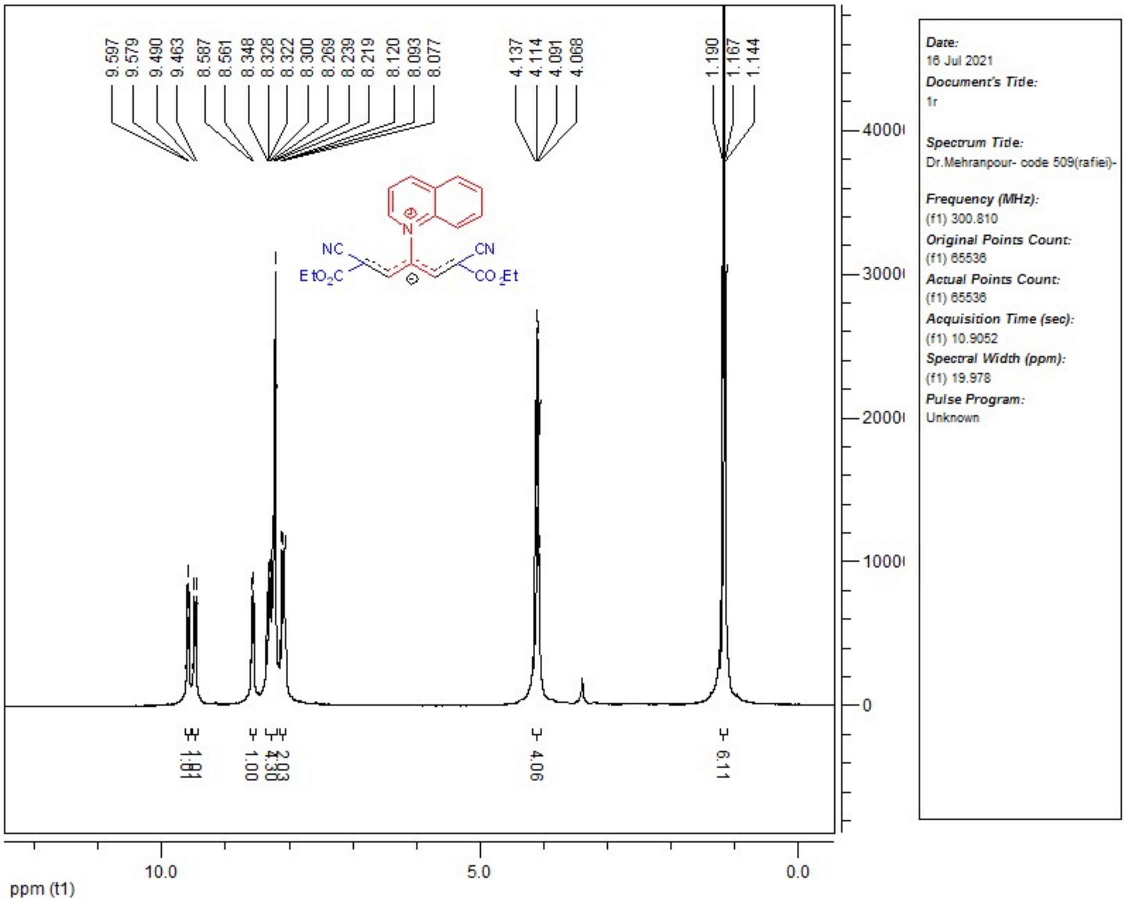
9.597
9.579
9.490
9.463
8.587
8.561
8.348
8.328
8.322
8.300
8.269
8.239
8.219
8.120
8.093
8.077
4.137
4.114
4.091
4.068
1.190
1.167
1.144
NC
CN
EtO2C
CO2Et
N
1.00
4.06
6.11
10.0
5.0
0.0
ppm (t1)
40000
30000
20000
10000
0
Date:
16 Jul 2021
Document's Title:
1r
Spectrum Title:
Dr.Mehranpour- code 509(rafiei)-
Frequency (MHz):
(f1) 300.810
Original Points Count:
(f1) 65536
Actual Points Count:
(f1) 65536
Acquisition Time (sec):
(f1) 10.9052
Spectral Width (ppm):
(f1) 19.978
Pulse Program:
Unknown

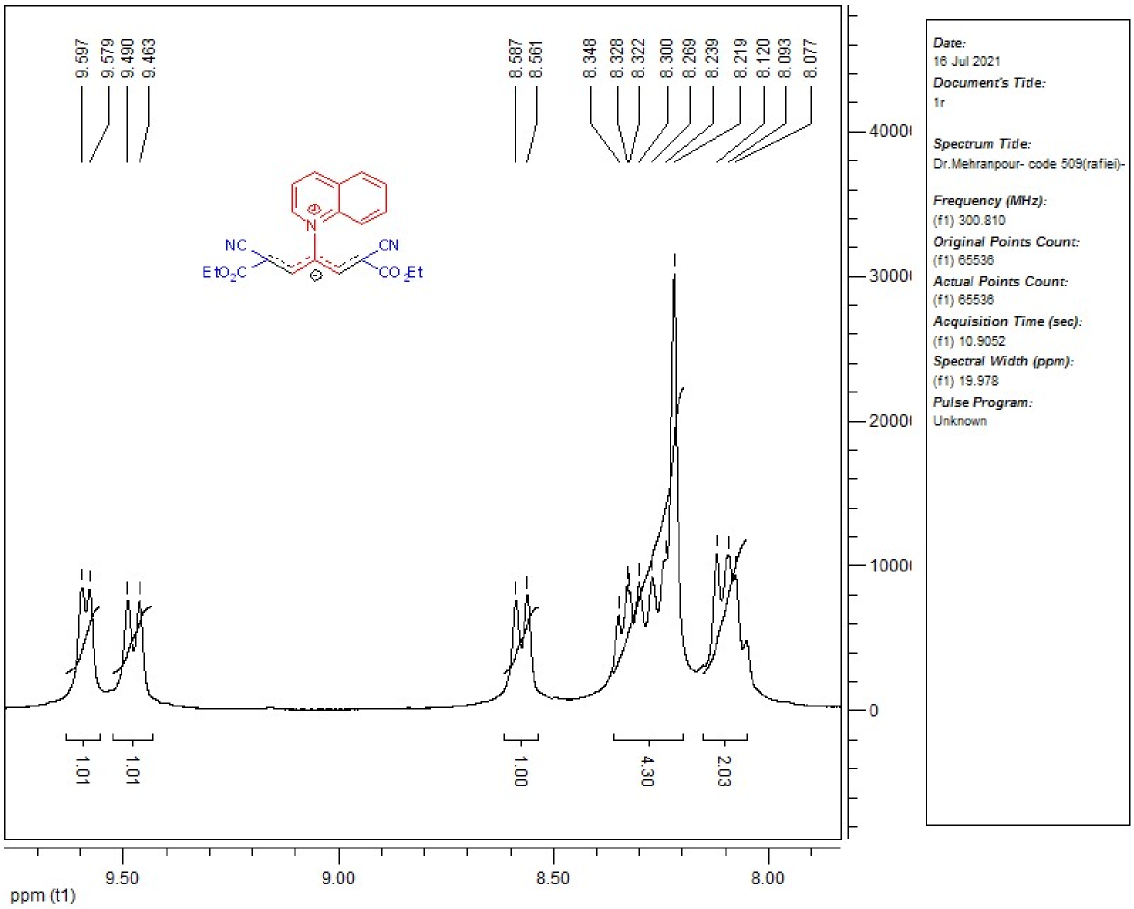
9.597
9.579
9.490
9.463
8.587
8.561
8.348
8.328
8.322
8.300
8.269
8.239
8.219
8.120
8.093
8.077
NC
CN
EtO2C
CO2Et
N
1.01
1.01
1.00
4.30
2.03
9.50
9.00
8.50
8.00
ppm (t1)
40000
30000
20000
10000
0
Date:
16 Jul 2021
Document's Title:
1r
Spectrum Title:
Dr.Mehranpour- code 509(rafiei)-
Frequency (MHz):
(f1) 300.810
Original Points Count:
(f1) 65536
Actual Points Count:
(f1) 65536
Acquisition Time (sec):
(f1) 10.9052
Spectral Width (ppm):
(f1) 19.978
Pulse Program:
Unknown

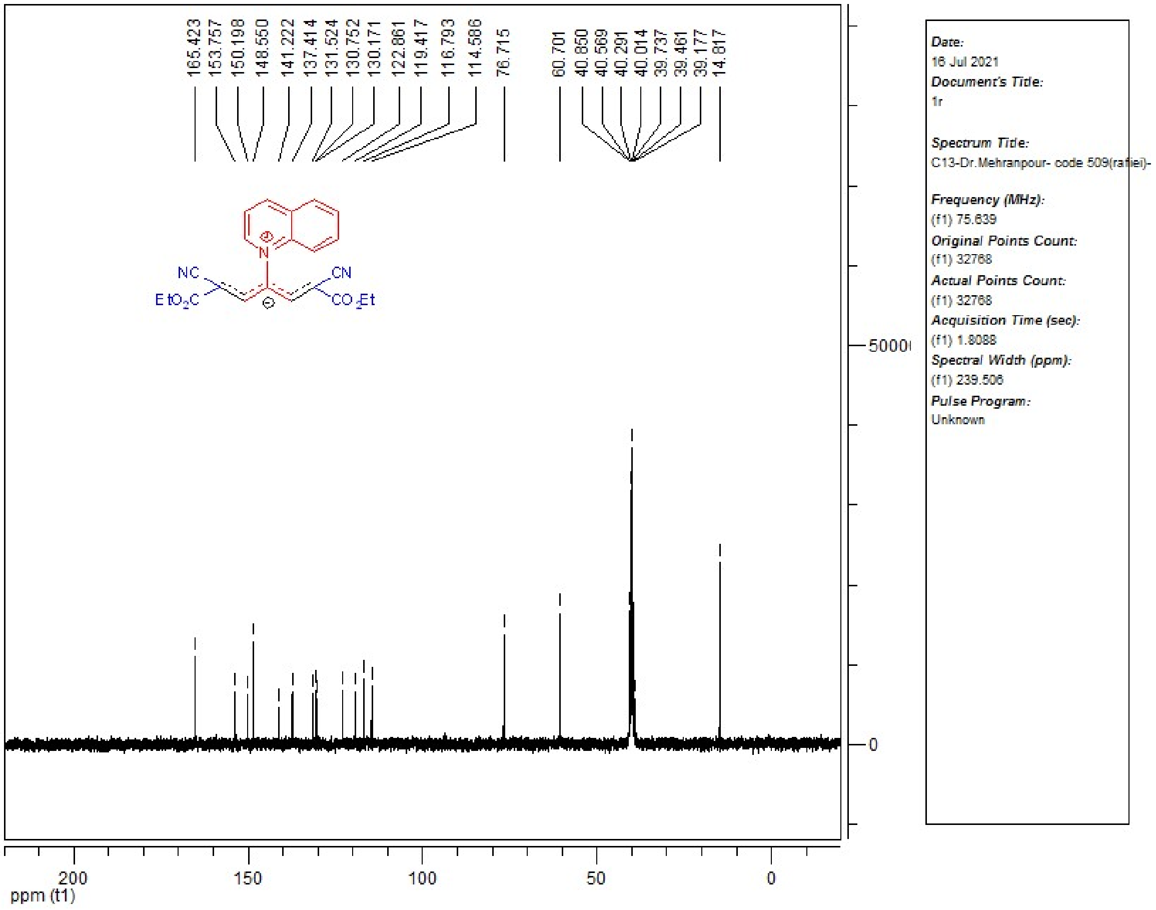

1,1,5,5-tetracyano-3-(isoquinolinium-2-yl)penta-propenides (4g):

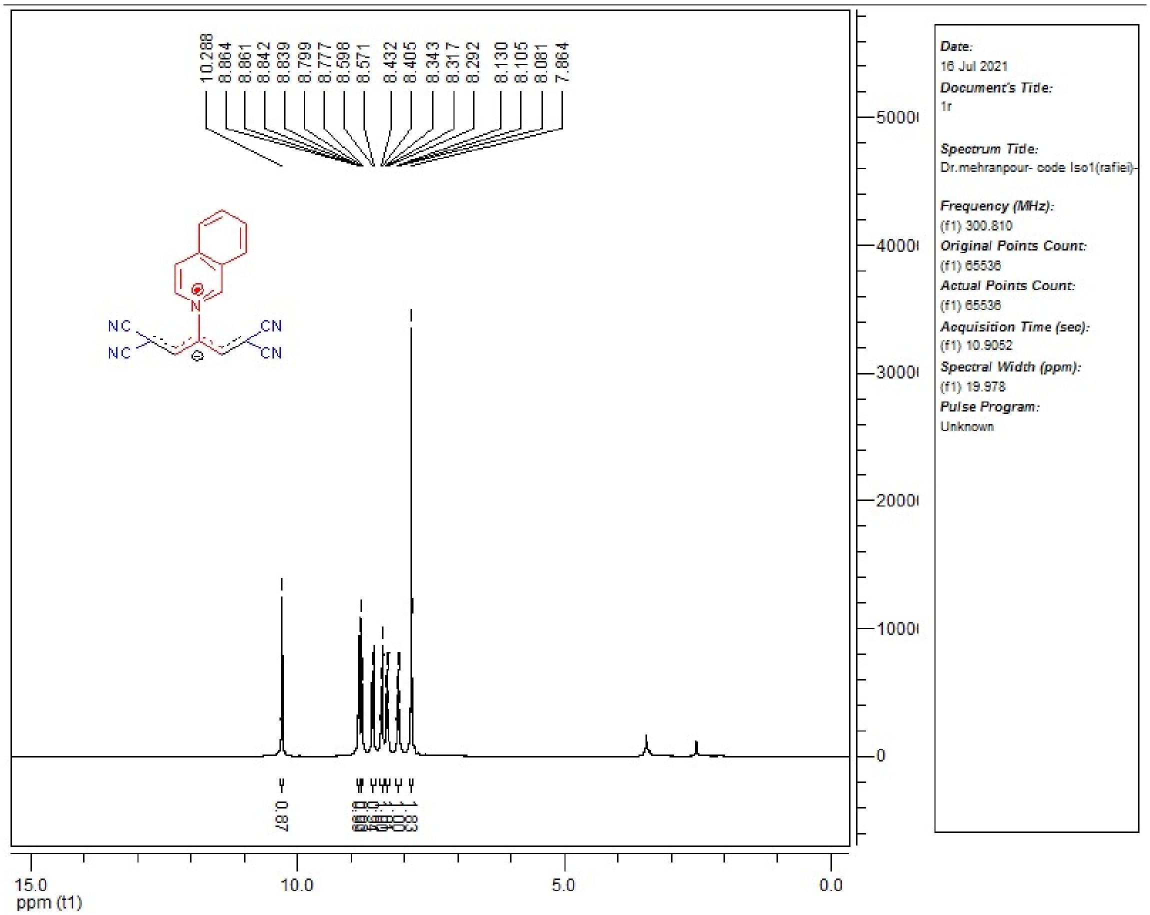
10.288
8.864
8.861
8.842
8.839
8.799
8.777
8.598
8.571
8.432
8.405
8.343
8.317
8.292
8.130
8.105
8.081
7.864
NC
NC
CN
CN
N
0.87
15.0
10.0
5.0
0.0
ppm (t1)
50000
40000
30000
20000
10000
0
Date:
16 Jul 2021
Document's Title:
1r
Spectrum Title:
Dr.mehranpour- code Iso1(rafiei)-
Frequency (MHz):
(f1) 300.810
Original Points Count:
(f1) 65536
Actual Points Count:
(f1) 65536
Acquisition Time (sec):
(f1) 10.9052
Spectral Width (ppm):
(f1) 19.978
Pulse Program:
Unknown

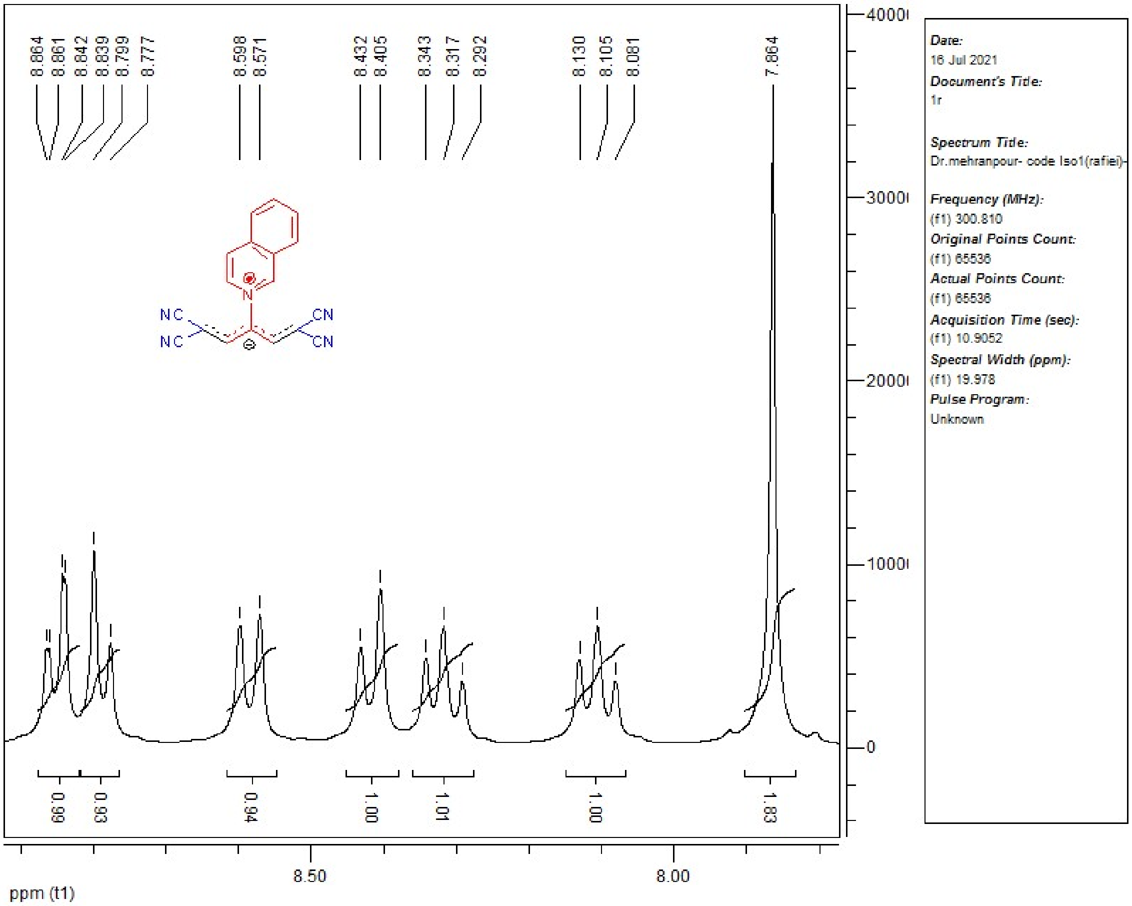

8.864
8.861
8.842
8.839
8.799
8.777
8.598
8.571
8.432
8.405
8.343
8.317
8.292
8.130
8.105
8.081
7.864
NC
NC
CN
CN
N
0.99
0.93
0.94
1.00
1.01
1.00
1.83
8.50
8.00
ppm (t1)
40000
30000
20000
10000
0
Date:
16 Jul 2021
Document's Title:
1r
Spectrum Title:
Dr.mehranpour- code Iso1(rafiei)-
Frequency (MHz):
(f1) 300.810
Original Points Count:
(f1) 65536
Actual Points Count:
(f1) 65536
Acquisition Time (sec):
(f1) 10.9052
Spectral Width (ppm):
(f1) 19.978
Pulse Program:
Unknown

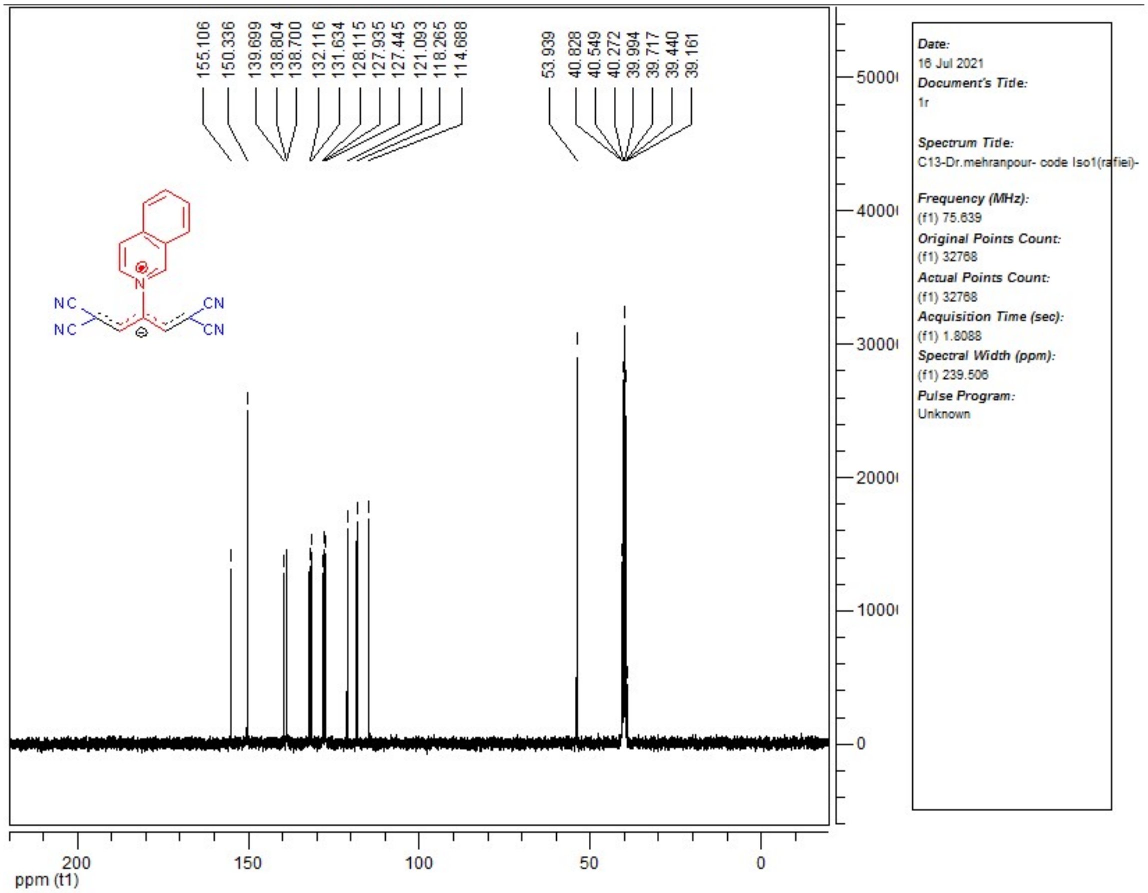
155.106
150.336
139.699
138.804
138.700
132.116
131.634
128.115
127.935
127.445
121.093
118.265
114.688
53.939
40.828
40.549
40.272
39.994
39.717
39.440
39.161
NC
NC
CN
CN
N
50000
40000
30000
20000
10000
0
200
150
100
50
0
ppm (t1)
Date:
16 Jul 2021
Document's Title:
1r
Spectrum Title:
C13-Dr.mehranpour- code Iso1(rafiei)-
Frequency (MHz):
(f1) 75.639
Original Points Count:
(f1) 32768
Actual Points Count:
(f1) 32768
Acquisition Time (sec):
(f1) 1.8088
Spectral Width (ppm):
(f1) 239.506
Pulse Program:
Unknown

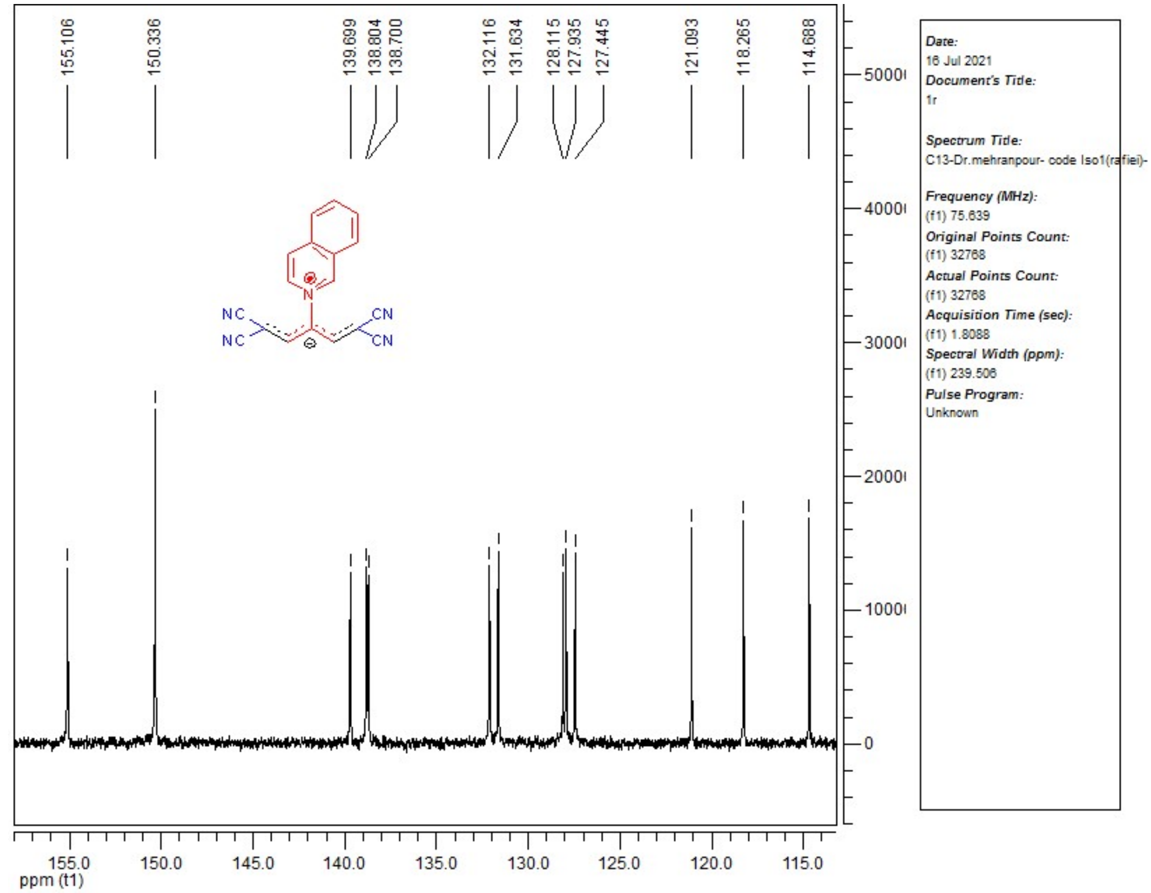

2,6-dicyano-1,7-diethoxy-4-(isoquinolinium-2-yl)-1,7-dioxo-penta-propenides (4h):

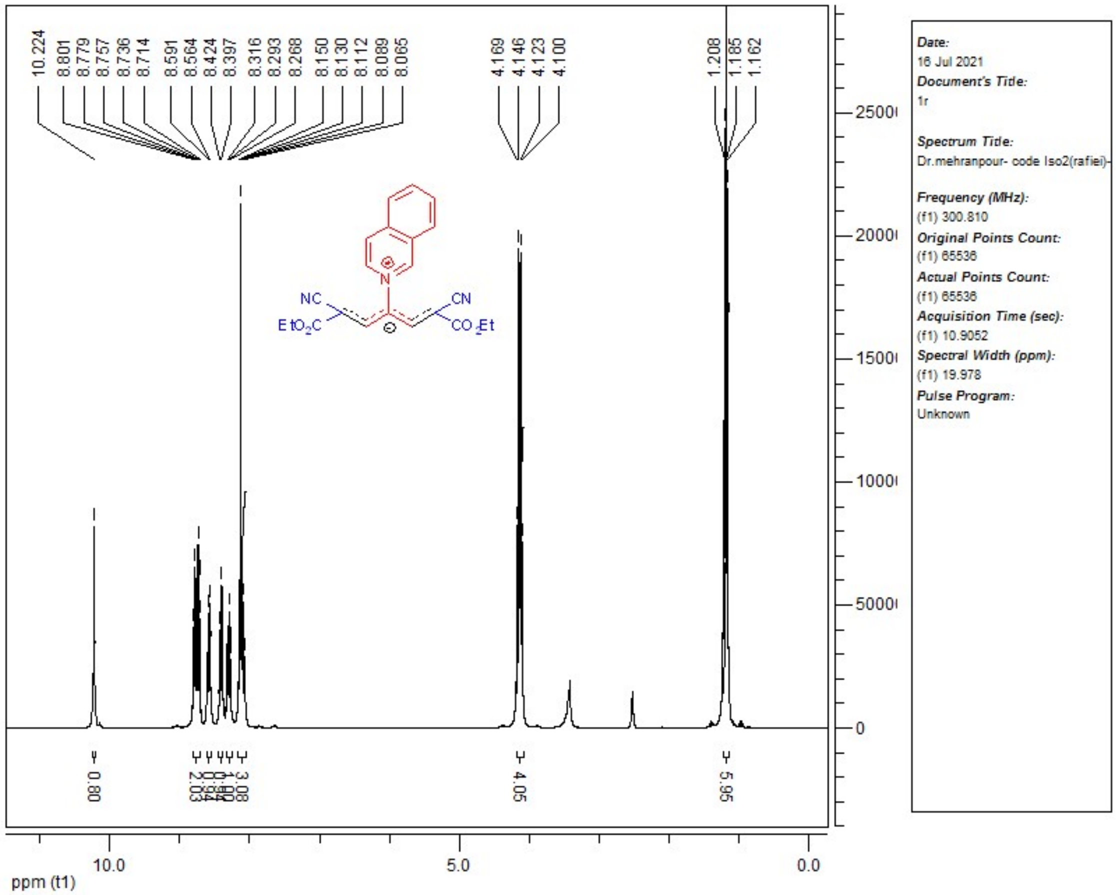
10.224
8.801
8.779
8.757
8.736
8.714
8.591
8.564
8.424
8.397
8.316
8.293
8.268
8.150
8.130
8.112
8.089
8.065
4.169
4.146
4.123
4.100
1.208
1.185
1.162
NC
CN
EtO2C
CO2Et
N
0.80
2.03
3.08
4.05
5.95
25000
20000
15000
10000
5000
0
10.0
5.0
0.0
ppm (t1)
Date:
16 Jul 2021
Document's Title:
1r
Spectrum Title:
Dr.mehranpour- code Iso2(rafiei)-
Frequency (MHz):
(f1) 300.810
Original Points Count:
(f1) 65536
Actual Points Count:
(f1) 65536
Acquisition Time (sec):
(f1) 10.9052
Spectral Width (ppm):
(f1) 19.978
Pulse Program:
Unknown

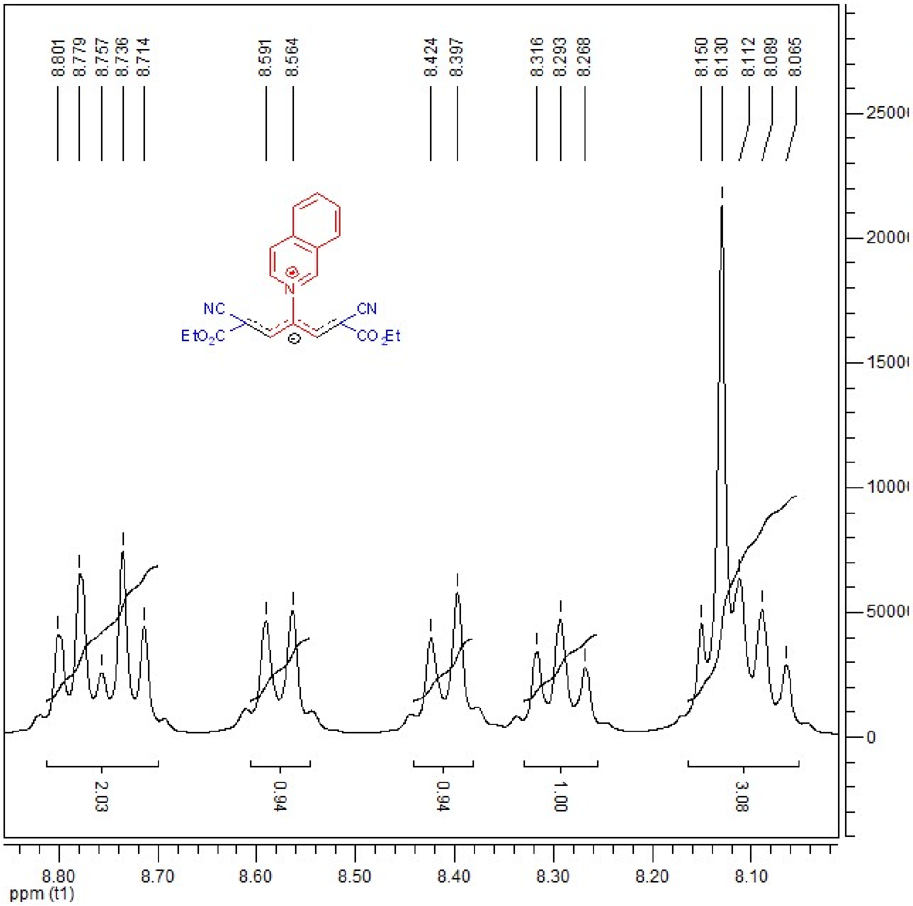

Date:
16 Jul 2021
Document's Title:
1r

Spectrum Title:
Dr.mehranpour- code Iso2(rafiei)-
Frequency (MHz):
(f1) 300.810
Original Points Count:
(f1) 65536
Actual Points Count:
(f1) 65536
Acquisition Time (sec):
(f1) 10.9052
Spectral Width (ppm):
(f1) 19.978
Pulse Program:
Unknown

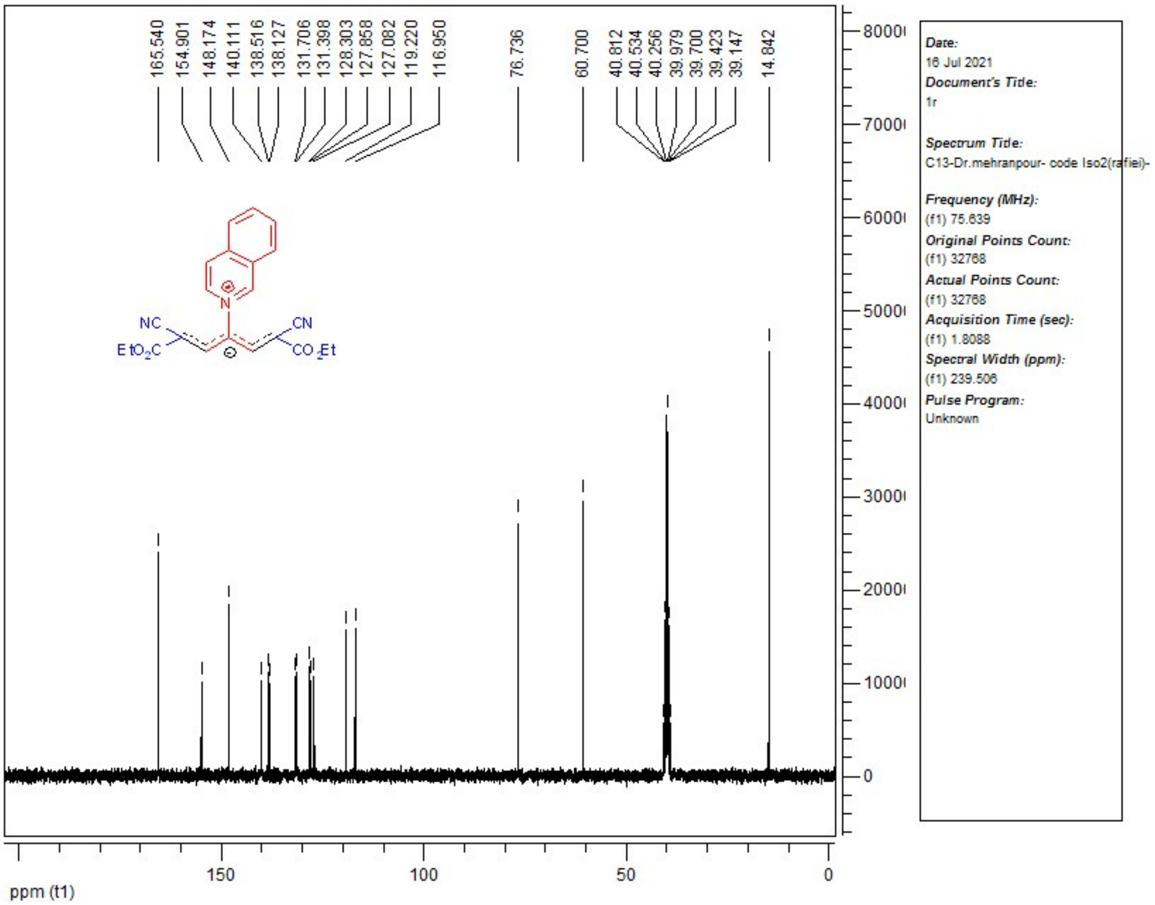
165.540
154.901
148.174
140.111
138.516
138.127
131.706
131.398
128.303
127.858
127.082
119.220
116.950
76.736
60.700
40.812
40.534
40.256
39.979
39.700
39.423
39.147
14.842
NC
CN
EtO2C
CO2Et
N
80000
70000
60000
50000
40000
30000
20000
10000
0
150
100
50
0
ppm (t1)
Date:
16 Jul 2021
Document's Title:
1r
Spectrum Title:
C13-Dr.mehranpour- code Iso2(rafiei)-
Frequency (MHz):
(f1) 75.639
Original Points Count:
(f1) 32768
Actual Points Count:
(f1) 32768
Acquisition Time (sec):
(f1) 1.8088
Spectral Width (ppm):
(f1) 239.506
Pulse Program:
Unknown

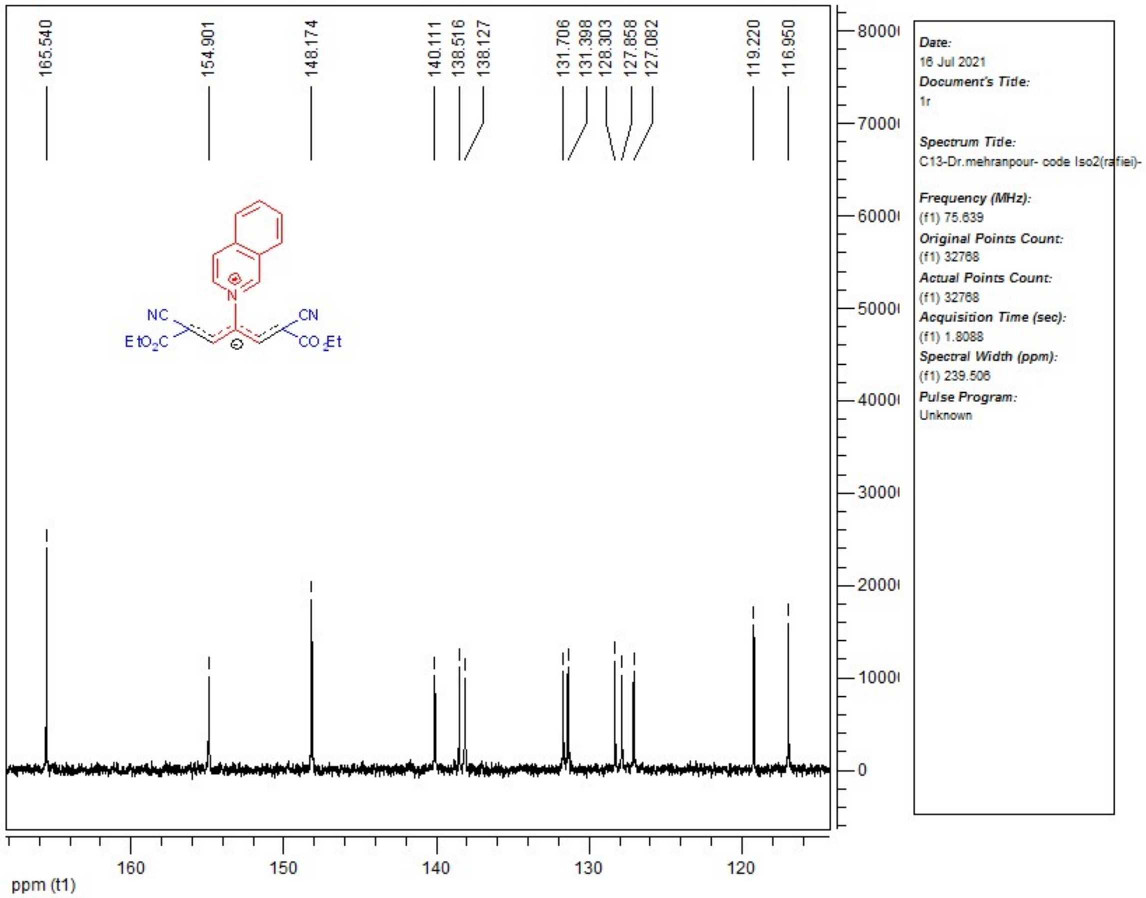
165.540
154.901
148.174
140.111
138.516
138.127
131.706
131.398
128.303
127.858
127.082
119.220
116.950
NC
CN
EtO2C
CO2Et
N
80000
70000
60000
50000
40000
30000
20000
10000
0
160
150
140
130
120
ppm (t1)
Date:
16 Jul 2021
Document's Title:
1r
Spectrum Title:
C13-Dr.mehranpour- code Iso2(rafiei)-
Frequency (MHz):
(f1) 75.639
Original Points Count:
(f1) 32768
Actual Points Count:
(f1) 32768
Acquisition Time (sec):
(f1) 1.8088
Spectral Width (ppm):
(f1) 239.506
Pulse Program:
Unknown
